# Working mechanism of a multidimensional computerized adaptive test for fatigue in rheumatoid arthritis

**DOI:** 10.1186/s12955-015-0215-7

**Published:** 2015-02-21

**Authors:** Stephanie Nikolaus, Christina Bode, Erik Taal, Harald E Vonkeman, Cees AW Glas, Mart AFJ van de Laar

**Affiliations:** Department of Psychology, Health and Technology, University of Twente, P.O. Box 217, 7500 AE Enschede, The Netherlands; Department of Research Methodology, Measurement and Data Analysis, University of Twente, P.O. Box 217, 7500 AE Enschede, The Netherlands; Medical Spectrum Twente, Department of Rheumatology and Clinical Immunology, P.O. Box 50 000, 7500 KA Enschede, The Netherlands; Expert Center for Chronic Fatigue, Radboud University Medical Center, P.O. Box 9101, 6500 HB Nijmegen, The Netherlands

**Keywords:** Multidimensional item response theory, Fatigue, Multidimensional computerized adaptive test, Item selection, Rheumatoid arthritis

## Abstract

**Background:**

This paper demonstrates the mechanism of a multidimensional computerized adaptive test (CAT) to measure fatigue in patients with rheumatoid arthritis (RA). A CAT can be used to precisely measure patient-reported outcomes at an individual level as items are consequentially selected based on the patient’s previous answers. The item bank of the CAT Fatigue RA has been developed from the patients’ perspective and consists of 196 items pertaining to three fatigue dimensions: severity, impact and variability of fatigue.

**Methods:**

The CAT Fatigue RA was completed by fifteen patients. To test the CAT’s working mechanism, we applied the flowchart-check-method. The adaptive item selection procedure for each patient was checked by the researchers. The estimated fatigue levels and the measurement precision per dimension were illustrated with the selected items, answers and flowcharts.

**Results:**

The CAT Fatigue RA selected all items in a logical sequence and those items were selected which provided the most information about the patient’s individual fatigue. Flowcharts further illustrated that the CAT reached a satisfactory measurement precision, with less than 20 items, on the dimensions severity and impact and to somewhat lesser extent also for the dimension variability. Patients’ fatigue scores varied across the three dimensions; sometimes severity scored highest, other times impact or variability. The CAT’s ability to display different fatigue experiences can improve communication in daily clinical practice, guide interventions, and facilitate research into possible predictors of fatigue.

**Conclusions:**

The results indicate that the CAT Fatigue RA measures precise and comprehensive. Once it is examined in more detail in a consecutive, elaborate validation study, the CAT will be available for implementation in daily clinical practice and for research purposes.

**Electronic supplementary material:**

The online version of this article (doi:10.1186/s12955-015-0215-7) contains supplementary material, which is available to authorized users.

## Background

Many patients with rheumatoid arthritis (RA) experience fatigue [1]. RA is a chronic auto-immune condition that is characterized by inflammation of the joints [2]. Typical symptoms besides fatigue are tender and swollen joints, pain, stiffness and functional limitations. Patients report far-reaching consequences of fatigue for daily life on a physical, emotional and social level. This symptom can have a negative impact on their ability to perform daily activities [1]. Fatigue in RA is different from normal tiredness, as it is often more extreme, not necessarily due to high levels of activity and, therefore, unpredictable [1,3-5].

To gain a better understanding of the causes of fatigue in RA and to provide adequate support to patients, it is essential to be able to accurately measure fatigue. There are already several uni- and multidimensional questionnaires in use to assess fatigue in clinical practice and research. Unidimensional questionnaires are usually brief and provide a single score, whereas multidimensional scales comprise a larger number of items and provide more detailed information that can give insights into different fatigue profiles and possibly into underlying fatigue mechanisms [6]. In line with patients’ experiences, fatigue should be measured on a multidimensional scale.

Most of the existing fatigue questionnaires were not specifically developed for an RA population. It remains debatable how appropriate these questionnaires are for measuring fatigue in RA. Generic fatigue items might be confounded by disease specific conditions such as disability or disease activity [7]. Moreover, the fatigue scales that are frequently used are traditional, fixed-length questionnaires, meaning that each patient has to fill in the same items in the same order. This method has the disadvantage that patients might be confronted with questions that do not match their individual level of fatigue. In contrast, a computerized adaptive test (CAT) is able to tailor measurements to the individual level of a patient. Items are selected from a large item bank, based on the patient’s previous answer. This leads to increased measurement precision, with fewer items than would be needed in traditional questionnaires [8]. Taking advantage of this modern measurement technology, we developed a multidimensional computerized adaptive test (CAT) based on the patients’ perspective for fatigue in RA, the “CAT Fatigue RA”.

Primarily, CATs are used for ability and achievement testing, but interest in computerized adaptive testing for health-related measures is growing. Research has already demonstrated that unidimensional CATs for depression and anxiety [9] and multidimensional CATs for dyspnoea and quality of life assessment [10,11] are efficient measurement instruments. The multidimensionality of our CAT Fatigue RA and its development from the patients’ perspective are novel aspects that differ from already existing CATs for fatigue such as the PROMIS CAT [12]. With a multidimensional instrument that calculates separate scores for each dimension of fatigue, it is possible to gain insight into the different fatigue profiles of each patient. Such insight can improve communication about fatigue in daily clinical practice, guide self-management strategies and interventions, and facilitate research into possible predictors of fatigue [13].

In this paper, we demonstrate the working mechanism of the item selection procedure by illustrating the course of the estimation of fatigue on each of the dimensions by using flowcharts and showing the items and answers per patient. Thereby, we propose the flowchart-check-method to control the multidimensional adaptive testing procedure of a CAT. According to our knowledge, this is the first detailed study into the working mechanism of a multidimensional CAT for the measurement of patient-reported outcomes.

**Figure 1:**
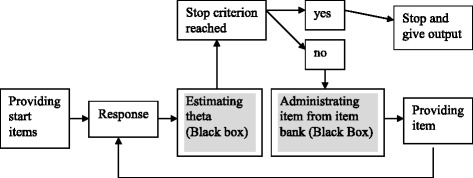
**Adaptive item selection procedure in CAT.**

For the development of a CAT, a large item bank is needed that contains far more items than can be presented to a single patient. For the computerized selection of the best matching items, precise information about the item characteristics is also needed. Item response theory (IRT) allows researchers to scale the item bank and independently determine item parameters, such as their difficulty level [8]. Each item reflects a level of fatigue, and all the items can be placed on a continuum, ranging from no fatigue to severe fatigue. Ideally, an item bank contains several items for each location on this continuum so that all levels of fatigue can be measured with high precision. Furthermore, IRT allows researchers to calculate how well an item discriminates between more or less fatigued patients. This information is required to match the items to the patient’s individual level and supports inter-individual comparisons, even when patients filled in different items. The level of fatigue is expressed in theta values, which is the usual entity in IRT and CAT for the estimation of the construct under consideration. Theta values are expressed on a metric with a mean of zero and a standard deviation of 1 [14]. In our study, a higher theta indicated a higher level of fatigue. The general adaptive item selection procedure of CATs is illustrated in Figure 1.

The adaptive item selection procedure of a CAT begins with the administration of pre-selected or randomly chosen start items. The responses to these start items serve as basis for a first estimation of theta. Based on this theta value, the CAT then selects as the next item one that has the greatest potential for reducing the uncertainty about the real theta of the patient. This next item is then provided and, based on the response, once again the next item with the greatest potential for reducing the uncertainty about the real theta is selected, and so on. This mechanism continues until a previously formulated stop criterion is reached. The CAT then stops and provides the final theta value.

In a multidimensional CAT, the item selection procedure is more complicated as items from different dimensions have to be selected. However, multidimensional adaptive testing offers equal or even higher precision with approximately one-third fewer items than would be needed in a unidimensional adaptive test [15]. Compared to a unidimensional CAT, a multidimensional CAT has several advantages. It provides separate estimates of fatigue on each dimension. The cross-information gained from items of correlated dimensions facilitates the CAT in its selection of the most informative items and estimation of fatigue with optimal precision. With this innovative method, measuring fatigue in RA can become more precise and, at the same time, more user-friendly.

The item selection process is based on algorithms programmed into the software and is not visible to the patient, as indicated in Figure 1 by the grey-shaded fields. This article illustrates the working mechanism of our CAT in a sample of patients with RA by showing each administered item together with the related response, theta and measurement precision per dimension and per person. The flowchart-check-method enabled us to control the adaptive item selection procedure. This study also investigated how a satisfactory measurement precision was reached on each dimension and whether the improvement in precision proceeded in a monotone way as would be the case for a unidimensional CAT. Moreover, the topic of CAT was vividly demonstrated and explained through the flowcharts depicting how the multidimensional mechanism of the CAT Fatigue RA worked and how the patients’ fatigue scores were generated.

## Method

### Participants and procedure

Participants were selected from a sample of patients who had participated in a previous study [16]. All participants in the previous study who had indicated interest in its results received a thank-you letter with information about the study outcomes. At the end of this letter, patients were informed about future studies and invited to register via e-mail if they wished to further participate. Once registered, the patient received an e-mail with detailed information about the new study. In addition, each patient was asked to agree to receive a telephone call from the first author in order to arrange for an appointment. Patients then participated in an individual session with the first author. Except for one appointment at the patient’s home, the sessions took place at the university. After receiving information about the study, patients signed an informed consent form and completed the measures described in this section. Travel costs for participants were refunded. This study was approved by the ethical review board of the University of Twente.

Six men and nine women diagnosed with RA were willing to participate. Mean age was 56.13 years (SD = 10.82), ranging from 35 to 71 years. All education levels were represented in the sample. Six participants were employed and nine participants were retired/disabled, homemakers or unemployed. The mean disease duration was 12.40 years (SD = 7.18), ranging from 3 to 24 years. About one third of the participants had co-morbidities. All sample characteristics are summarized in Table 1.

**Table 1:** **Sample characteristics (N = 15)**

	**N/Mean (S.D.)**	**Range**
Gender		
Women	9	
Men	6	
Level of education		
Lower (≤12 years of education)	4	
Average (13–14 years of education)	6	
Higher (>14 years of education)	5	
Work status		
Working full-time	4	
Working part-time	2	
Household/unemployed	2	
Disabled/Retired	7	
Co-morbidities		
Yes	4	
No	9	
Age (years)	56.13 (10.82)	35 - 71
RA Disease duration (years)	12.40 (7.18)	3 - 24
NRS Global health	4.27 (2.12)	0 - 7
NRS Pain	4.20 (2.46)	0 - 8
NRS Fatigue	5.80 (2.18)	1 - 9

NRS = Numerical Rating Scale; RA = rheumatoid arthritis.

### Measures

All participants completed a background questionnaire that included items about gender, age, education, work status, as well as disease-specific information: disease duration, co-morbidity, numerical rating scale (NRS) global health, pain and fatigue. The NRSs had eleven points (ranging from 0 to 10) and the following anchors: very good/very poor, no pain/unbearable pain, no fatigue/totally exhausted. The patients then filled in the CAT on a computer. The CAT was presented within the web system ROMA (Rheumatology Online Monitor Application) that is currently used by the Arthritis Centre Twente. Patients received one question per screen. Once selecting their answer, they then clicked on a button to receive the next question.

### The CAT Fatigue RA

The item pool was thoroughly developed from the patients’ perspective and evaluated in several steps. To capture all relevant aspects of fatigue, first the experience of fatigue was investigated [6,17]. Then items and dimensions of existing fatigue scales were collected in a preliminary item pool and supplemented with items from interview material [6]. This item pool was evaluated in a Delphi study with experts (patients, nurses, and rheumatologists) to select adequate items to measure fatigue in RA [18-20]. The final content valid item pool consisted of 245 items and 12 dimensions. The dimensions were: severity, frequency, duration, changes in fatigue, perceived causes of fatigue, energy, sleep/rest, body feeling, cognition/concentration, coping, negative emotions/mood, and consequences. In a consecutive study, how well the items fit with pre-defined dimensions was assessed, and the item pool’s dimensionality structure was examined in statistical terms [16]. For this assessment, IRT analyses were used, and finally a between-items multidimensional IRT-model was fitted to the data.

The initially calibrated item bank that was used for the development of the CAT Fatigue RA consisted of 196 items and 3 dimensions, which included all 12 dimensions that emerged from the Delphi study. These items and dimensions were: 13 items representing severity (covering the dimensions of severity, duration, and frequency), for example: Did you feel tired during the last 7 days?; 169 items representing impact (covering the dimensions of cognition/concentration, negative emotions/mood, energy, sleep/rest, body feeling, coping, and consequences), for example, Have you felt down or dejected because of fatigue? During the past 7 days, I was too tired to do my most important tasks; and 14 items representing variability (covering the dimensions of change and perceived causes), for example: How did your fatigue change during the last 7 days?

The algorithm of the CAT was constructed according to Segall’s [15] work on multidimensional adaptive testing. In multidimensional CATs, item selection is based on Bayesian principles. That means that the item which has the greatest potential to reduce the statistical uncertainty about the fatigue level of the patient is selected from the potential items in the bank. This process is aided by information about the correlations between the dimensions [15]. The precise correlations and also the item parameters that resulted from the previous calibration study are reported elsewhere [16].

Each participant answered twenty items before the CAT stopped automatically. The CAT started with two random start items per dimension and always administered at least five items per dimension. These characteristics of the CAT were based on simulations with approximately 1000 virtual patients. For the current version of the CAT Fatigue RA, this combination of twenty items in total, two start items and five items per dimension was the most optimal solution in terms of test-length and measurement error for each dimension.

### Analyses

The data of the background questions were entered into SPSS 20 and descriptive statistics were calculated. Data of the CAT were stored on a server and could be downloaded by the researchers in the form of Excel files. In the Excel files, the following data were stored: each administered item, the given answer, the estimated level of fatigue (theta-value) on each of the three dimensions and the respective standard error on each of the three dimensions. In the “Results” section, these data are shown for each of the fifteen participants. The five most interesting examples are included in Additional file 1: Examples 5, 6, 9, 10 and 12; the other ten examples are available as an Additional file 2. First, two flowcharts per patient illustrate the course of the estimation of the fatigue level and the decrease of the standard error during the CAT administration. Second, theta-values and standard errors as well as selected items and given answers are shown in tables.

1. Check of the CAT’s working mechanism
  The flowcharts, thetas, standard errors and items with answers served as basis for a check of the working mechanism of the CAT Fatigue RA. The flowcharts indicated whether the adaptive test procedure followed a logical sequence and whether items and answers were in accordance with the theta estimate. For each of the fifteen CAT administrations, the researchers analysed all twenty theta values on the three dimensions while also examining the provided items and given answers to determine whether the theta value developed in accordance to the answers. For example, if a patient filled in the highest possible answer category, representing a high level of fatigue, it was expected that the following theta would increase or at least remain at the same level compared to the previous theta value.
2. Item selection in the CAT
  For each patient, the researchers examined which items had been selected. They registered to which dimension each item pertained and in which sequence the items from the different dimensions were selected. The researchers also determined whether the administration rules had been followed; that is, two random start items per dimension and at least five items per dimension.
3. Measurement precision
  For each of the 15 CAT administrations, the measurement precision of each of the three dimensions was investigated. The researchers went through the twenty standard error values per dimension to check the precise course of the measurement precision. Measurement precision was graphically illustrated in the flowcharts, and lines were placed in these charts to highlight the point at which an acceptable measurement precision had been reached.
  This measure-precision analysis was completed in-line with the following rationale.
  The standard error refers to the theta estimation. A standard error ≤ 0.32 is regarded as an indicator for satisfactory reliability of a CAT, corresponding to a reliability of r = 0.90 [21]. This reliability criterion has also been adopted for existing CATs, for example, when measuring depression or stress [22,23]. For the CATs in these previous studies, however, the researchers used a flexible stopping rule, which meant that the administration of the CAT was not determined by a fixed number of items, unlike the CAT Fatigue RA, but by reaching an acceptable low standard error, leading to variable test-lengths. Moreover, the CATs in these previous studies were unidimensional. Nevertheless, the criterion of 0.32 can also be used for the evaluation of the measurement precision of a multidimensional CAT. Since the theta-distribution on each dimension has a standard deviation of 1, the standard error of 0.32 entails a proportion of true variance of 0.90, which is analogous to the unidimensional case. In other words, the criterion can be applied to each of the dimensions separately.
  In the standard error flowcharts, a vertical solid line indicates the point where the standard error have reached a level ≤ 0.32 on all three dimensions. A dashed line indicates that standard errors ≤ 0.32 were reached for the dimensions severity and impact and that the standard errors of the dimension variability did not show variations larger than 0.05 until the end of the CAT administration.
4. Scores of fatigue
  Whether different scoring patterns were present in the sample was also examined. For each patient, the final fatigue scores on the three dimensions were checked and sorted in descending order, according to their size. All different combinations (for example, highest score on the dimension severity, middle score on impact and the lowest score on variability) were registered.

## Results

In this section, we present the results of the item selection procedure of the multidimensional CAT Fatigue RA. Five examples (Additional file 1) were chosen to demonstrate as much diversity as possible and to illustrate the most interesting findings regarding different numbers of items per dimension, different courses of the standard error per dimension and different scoring patterns on the three dimensions per participant. The other ten examples can be found online as Additional file 2.

1. Check of the CAT’s working mechanism
  As displayed in the flowcharts, the administered items and given answers were thoroughly compared with the course of the theta estimation, and no logical flaws were identified. These results showed that the theta values were not higher or lower than the researchers’ expectations which had been based on the content of the provided item and given answer. Consequently, this check served as a control for a correct working mechanism of the newly developed measurement instrument, and provided a first indication that the adaptive testing process works well. In addition, this check showed that the measurement was adapted to each patient and that the most informative items were selected. The flowcharts also showed that the information of the correlation between the dimension was taken into account when estimating the level of fatigue. After the adminstration of an item, the theta values not only changed on the dimension of the administered item, but also on the other two dimensions.
2. Item selection in the CAT
  The CAT always started with the administration of two random items of the severity dimension, two random items of the impact dimension, and two random items of the variability dimension, and then selected two further items of the variability dimension and at least two further items of the impact dimension. The selection of the remaining ten items was differentially distributed among the three dimensions. Hence, after answering item 10, patients received different numbers and sequences of items from the dimensions, according to their individual level of fatigue. The criterion of at least five items per dimension was always fulfilled, and the overall number of items per dimension varied per example. The allocation varied from 5 to 7 severity items, 8 to 10 impact items and 5 to 7 variability items.
3. Measurement precision
  In four of the fifteen examples, the standard error became ≤ 0.32 for each dimension before the CAT stopped at the stop criterion of twenty items (twice after item 9, once after item 15, and once after item 16). The situation of a standard error ≤ 0.32 for the first two dimensions and a clear decrease of the standard error on the third dimension emerged in all of the other examples between 6 and 17 administered items. The variability dimension only reached a measurement precision value ≤ 0.32 in four of the fifteen examples, whereas the other two dimensions reached a standard error ≤ 0.32 after, at most, 13 or rather 17 administered items (see Additional file 1: Example 10). However, for the variability dimension, the standard error also clearly decreased during the CAT administration. Even the highest final standard error on the variability dimension (0.4339) was equivalent to r = 0.81, reflecting good reliability [21,24].
  In Additional file 1: Example 10, satisfactory measurement precision was reached after 11 items, but after the administration of item 16, the standard error of the impact dimension increased to a level above 0.32 before it decreased again. Instead of a monotone decrease as expected in a unidimensional CAT, all the examples show a nonmonotone development of the standard errors on each of the three dimensions. This result is because the algorithm minimizes the volume of a three-dimensional reliability region, that is, a three-dimensional ellipse. Note, however, that such a minimalization is not equivalent to separately decreasing the reliability region on each dimension.
4. Scores of fatigue
  The flowcharts further illustrated that each patient had different fatigue scores, both at an individual level of fatigue, as well as different scores on the dimensions. This result was especially visible in the flowcharts of Additional file 1: Examples 6 and 9. For the most part, patients reached the highest theta on the dimension severity, then on impact and lastly on variability. But other combinations of scores also emerged in the examples, including from highest to lowest score: impact, severity, variability and variability, impact, severity.

## Discussion and conclusions

This study demonstrated the working-mechanism of the recently developed multidimensional CAT Fatigue RA. The adaptive item selection procedure with its course of scores and measurement precision has been described. The results showed the inner process of the CAT wherein the theta’s and standard errors for each dimension were estimated in relation to each other according to a multidimensional IRT model. Consequently, the selection of an item from one dimension influenced the estimation of the theta’s and standard errors of the other dimensions which led to nonmonotonic changes of the estimated standard errors over the number of applied items.

The correct working mechanism of the CAT was demonstrated. The CAT worked according to the pre-defined administration rules and had good to excellent measurement precision. Moreover, different scoring patterns on the three dimensions were found. The results of this study have implication for the further use and research of the CAT Fatigue RA, such as regarding the stopping rule and the variability dimension.

### Measurement precision and stopping rule

The standard errors on the dimension severity and impact reached an excellent level and on the dimension variability a satisfactory level before the end of the CAT administration. The standard errors remained relatively stable over the remaining items, indicating that they probably would not decrease further, even after administering more items. Consequently, administering less than twenty items or working with a flexible stopping rule as in unidimensional CATs is worth consideration [22,23]. With a flexible stopping rule at a standard error equal to or smaller than 0.32, in this study, some patients would only have had to fill in between 9 to 16 items, minimizing their burden and time investment. However, such a decision must be made with caution. In a multidimensional CAT, standard errors do not decrease monotonously as in a unidimensional CAT. The application of a stopping rule purely based on reaching a certain standard error could, therefore, lead to some discrepancies. In Example 10 of this study, even after the first fulfilment of the criterion for a satisfactory standard error, the standard error again increased after additional items. The reason for this phenomenon has been previously addressed in the Results section. Due to these results, future studies of the CAT Fatigue RA need to examine this issue in more detail and include a larger sample of patients.

### Quality of the variability dimension

The higher measurement error of the dimension variability compared to the dimensions severity and impact, was also reflected in the fact that the item selection always started with two variability items after the respective random start items had been completed. A CAT always selects the item that leads to the maximum increase in measurement precision. In other words, the CAT strives to maximally reduce the uncertainity of the measurement of a person’s fatigue level. Logically, the CAT should always administer more items of the variability dimension than of the other two dimensions. However, this was not the case perhaps due to the fact that the variability dimension did not include enough adequate items that measure on different locations of fatigue in the item pool. These finding correspond to the previous calibration study [16] where the variability dimension was shown to be less stable in psychometric terms than the other two dimensions. The variability dimension contains items about the changing character of fatigue along with items about reasons that patients attribute to their fatigue. It is, therefore, not surprising that the variability dimension had more measurement error than the dimensions severity and impact.

The quality of the variability dimension has to be examined in future studies. Larger sample sizes and a recalibration should give an answer to the question whether the variability dimension should remain included in future versions of the CAT Fatigue RA. It is important to note, however, that the variability dimension contains issues mentioned by the patients themselves, and these issues, to date, do not exist in fatigue questionaires to the same degree. Our aim was to build a CAT based on the perspective of patients, so in the development of the item bank [16], the optimal balance between both perspectives – psychometric results and information gained from patients’ experience – remained our goal. In order to protect the content validity of the CAT, we decided to let the CAT select at least five items per dimension. This decision ensured that items from the variability dimension were also drawn in the adaptive testing process, despite possibly having less optimal item characteristics.

### Scores on each fatigue dimension

The preservation of the variability dimension provided interesting information about the fatigue experience of patients. Even in this small sample, all possible combinations of scores on the three dimensions were present. Different fatigue experiences might be reflected when a patient scores especially high on severity versus on impact or variability of fatigue. However, it has to be noted that the different combinations of fatigue scores in this study were purely descriptive. For statistically sound conclusions, future analysis should include whether these differences between scores on the dimensions are significant. Nicklin et al. [13] also found differentially high scores on the Bristol Rheumatoid Arthritis Fatigue (BRAF) short scales for fatigue severity, fatigue effect and coping with fatigue. The severity and effect scales clearly showed higher correlations with each other than they each did with the coping scale. Nicklin et al. [13] pointed to the possibility of improving coping with fatigue and thus reducing its effect even when severity remained the same.

Based on the items of the dimension variability, clinicians and patients could gain more insight into the variability of the patients’ fatigue and the potential facilitators in their lives. Those patients scoring high on this dimension experience changes in fatigue and attribute certain factors to their fatigue. Such an understanding could possibly allow for greater personal communication about these issues with health professionals, allowing them to detect possibilities for the patients to improve their coping abilities or to adapt disfunctional cognitions about their fatigue.

### The flowchart-check-method

Finally, this study proposed the flowchart-check-method to investigate the working mechanism of a multidimensional CAT. To the best of our knowledge, no standard method for checking a multidimensional CAT is, as yet, available. The flowchart-check-method worked well and provided a significant amount of information about the adaptive working mechanism of our CAT. This study allowed us to examine the item selection procedure for the three dimensions in a sample of patients. The course of the measurement precision was also unraveled, providing important insights into the development of standard errors on the three separate dimensions. A disadvantage of this method might be the large amount of work needed to present detailed information per patient. Possibly a smaller number of examples, such as ten, would have also been sufficient to check the CAT’s working mechanism on a heterogeneous sample of patients. The main advantage, however, is that such a check enables researchers to determine whether a CAT operates as intended. In addition, researchers can recognize patterns in the score development and study the course of the measurement precision. Without this study, it would not have been possible to discover the non-monotone courses of the standard errors per dimension, which is essential information for future decisions on the stopping rule of the CAT. Furthermore, the flowchart-check-method allows to detect mistakes in the CAT’s working mechanism and to determine the programming accuracy. In summary, we believe that this study forms an essential part in the development and validation of a (multidimensional) CAT.

After investigating the measurement characteristics of the CAT Fatigue RA in a consecutive, elaborate validation study, we plan to make this new measurement instrument available for use in daily clinical practice and for research purposes. Aim is that the CAT Fatigue RA assesses fatigue more precisely, comprehensively and with fewer items than traditional questionnaires. Unraveling the adaptive test mechanism of a CAT can make this modern and complex technique more accessible to a broader public and facilitate its use in different scientific and practical areas.

## Additional files

Additional file 1:
**Example 5, Example 6, Example 9, Example 10, Example 12.** For each of the examples, the following information is provided. **Flowchart 1.** Course of the theta scores per fatigue dimension over the administered items. **Flowchart 2.** Course of the standard errors per fatigue dimension over the administered items. **Table S1.** Theta scores and standard errors per dimension for each of the administered items. **Table S2.** Administered items (order, dimension, number in item bank, text and given answer). Additional file 2:
**Example 1, Example 2, Example 3, Example 4, Example 7, Example 8, Example 11, Example 13, Example 14, Example 15.** For each of the examples, the following information is provided. **Flowchart 1.** Course of the theta scores per fatigue dimension over the administered items. **Flowchart 2.** Course of the standard errors per fatigue dimension over the administered items. **Table S1.** Theta scores and standard errors per dimension for each of the administered items. **Table S2.** Administered items (order, dimension, number in item bank, text and given answer).
