# Supplementary material for: Working mechanism of a multidimensional computerized adaptive test for fatigue in rheumatoid arthritis
Source: Health Qual Life Outcomes. 2015 Feb 21;13:23. doi: 10.1186/s12955-015-0215-7 (PMC4340497; doi:10.1186/s12955-015-0215-7)
Supplement: Additional file 1: — Example 5, Example 6, Example 9, Example 10, Example 12. For each of the examples, the following information is provided. Flowchart 1. Course of the theta scores per fatigue dimension over the administered items. Flowchart 2. Course of the standard errors per fatigue dimension over the administered items. Table S1. Theta scores and standard errors per dimension for each of the administered items. Table S2. Administered items (order, dimension, number in item bank, text and given answer). [file 12955_2015_215_MOESM1_ESM.doc]

**Flow-chart material**

Example 5


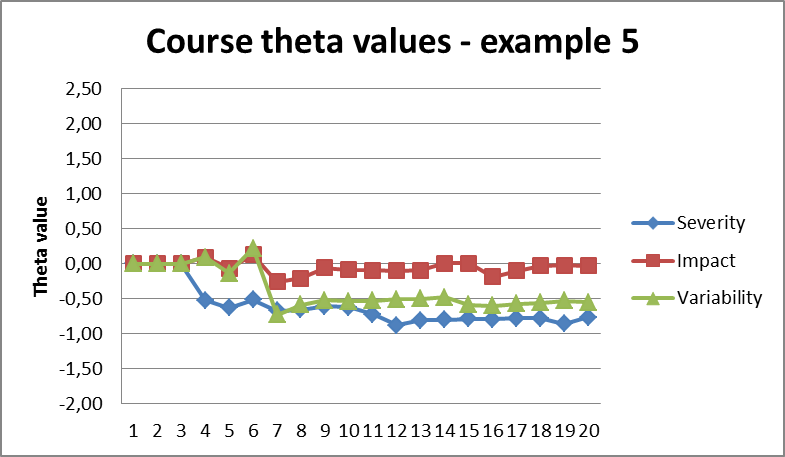


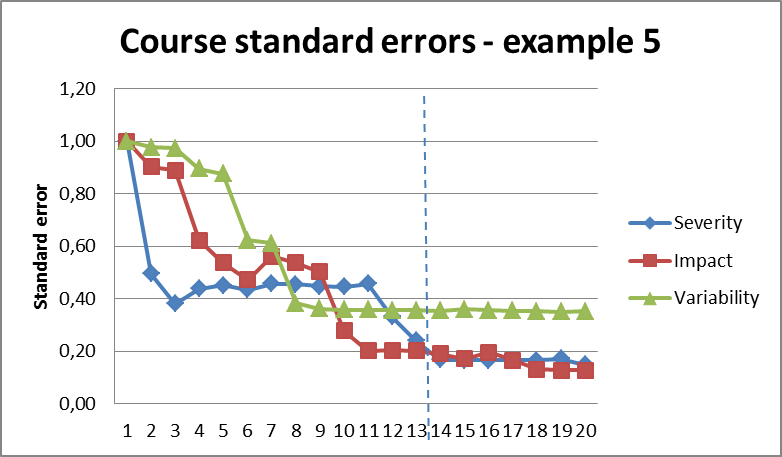


| item | | dimension | | | theta d1 | theta d2 | theta d3 | SE d1 | SE d2 | | SE d3 |
| --- | --- | --- | --- | --- | --- | --- | --- | --- | --- | --- | --- |
| 1 | | 1 | | | 0,0000 | 0,0000 | 0,0000 | 1,0000 | 1,0000 | | 1,0000 |
| 2 | | 1 | | | 0,0000 | 0,0000 | 0,0000 | 0,4939 | 0,9026 | | 0,9767 |
| 3 | | 2 | | | 0,0000 | 0,0000 | 0,0000 | 0,3803 | 0,8891 | | 0,9736 |
| 4 | | 2 | | | -0,5230 | 0,0876 | 0,0926 | 0,4376 | 0,6222 | | 0,8951 |
| 5 | | 3 | | | -0,6311 | -0,0675 | -0,1369 | 0,4498 | 0,5359 | | 0,8748 |
| 6 | | 3 | | | -0,5166 | 0,1253 | 0,2217 | 0,4341 | 0,4713 | | 0,6228 |
| 7 | | 3 | | | -0,6738 | -0,2592 | -0,7315 | 0,4563 | 0,5614 | | 0,6110 |
| 8 | | 3 | | | -0,6631 | -0,2107 | -0,5879 | 0,4545 | 0,5371 | | 0,3823 |
| 9 | | 2 | | | -0,6131 | -0,0563 | -0,5274 | 0,4472 | 0,5024 | | 0,3605 |
| 10 | | 2 | | | -0,6215 | -0,0917 | -0,5404 | 0,4447 | 0,2779 | | 0,3580 |
| 11 | | 1 | | | -0,7261 | -0,0972 | -0,5296 | 0,4573 | 0,2020 | | 0,3568 |
| 12 | | 1 | | | -0,8835 | -0,1038 | -0,5066 | 0,3299 | 0,2026 | | 0,3556 |
| 13 | | 1 | | | -0,8112 | -0,0950 | -0,5061 | 0,2392 | 0,2014 | | 0,3555 |
| 14 | | 2 | | | -0,8051 | 0,0048 | -0,4842 | 0,1663 | 0,1892 | | 0,3543 |
| 15 | | 3 | | | -0,7915 | 0,0038 | -0,5874 | 0,1655 | 0,1711 | | 0,3596 |
| 16 | | 2 | | | -0,7943 | -0,1859 | -0,6058 | 0,1657 | 0,1945 | | 0,3546 |
| 17 | | 2 | | | -0,7822 | -0,1024 | -0,5713 | 0,1649 | 0,1643 | | 0,3526 |
| 18 | | 2 | | | -0,7796 | -0,0311 | -0,5618 | 0,1648 | 0,1305 | | 0,3519 |
| 19 | | 1 | | | -0,8605 | -0,0272 | -0,5288 | 0,1698 | 0,1258 | | 0,3501 |
| 20 | | 1 | | | -0,7653 | -0,0285 | -0,5517 | 0,1473 | 0,1259 | | 0,3513 |
| item | dim. | | nr. | itemstam | | | | | | answer | |
| 1 | 1 | | 7 | I was particularly tired in the evening. | | | | | | 1-2 days | |
| 2 | 1 | | 4 | Fatigue was the worst consequence of my rheumatism. | | | | | | 2 | |
| 3 | 2 | | 80 | Have you felt you have less control about your life because of fatigue? | | | | | | a little | |
| 4 | 2 | | 181 | Fatigue caused me to become impatient when things didn´t succeed. | | | | | | not at all | |
| 5 | 3 | | 183 | To what degree has your fatigue changed? | | | | | | stayed the same | |
| 6 | 3 | | 189 | Performance of routine daily activities increases my fatigue. | | | | | | yes | |
| 7 | 3 | | 191 | Rheumatoid arthritis is the cause of my fatigue. | | | | | | no | |
| 8 | 3 | | 194 | Pain and / or other problems are the cause of my fatigue. | | | | | | yes | |
| 9 | 2 | | 138 | I really didn´t look forward to doing something due to the fatigue. | | | | | | a little | |
| 10 | 2 | | 38 | The fatigue felt like a physical burden. | | | | | | a little | |
| 11 | 1 | | 10 | I was even tired if I hadn´t done anything special. | | | | | | rarely | |
| 12 | 1 | | 1 | Please circle the number that shows your average level of fatigue. | | | | | | 1 | |
| 13 | 1 | | 8 | Did you feel fatigued? | | | | | | sometimes | |
| 14 | 2 | | 150 | Fatigue meant I was less able to perform well. | | | | | | a little | |
| 15 | 3 | | 186 | The severity of my fatigue could vary. | | | | | | no | |
| 16 | 2 | | 134 | I had to make choices which things I would or would not do, because I was too tired to do everything. | | | | | | not at all | |
| 17 | 2 | | 167 | Because of fatigue, I was less able to complete tasks that require physical effort. | | | | | | sometimes | |
| 18 | 2 | | 114 | I had to limit my social activities because I was tired. | | | | | | a little | |
| 19 | 1 | | 9 | How often have you been fatigued? | | | | | | occasionally but not most days | |
| 20 | 1 | | 11 | I soon tired whilst I was doing something. | | | | | | sometimes | |

All items had a time frame of the last 7 days

Example 6


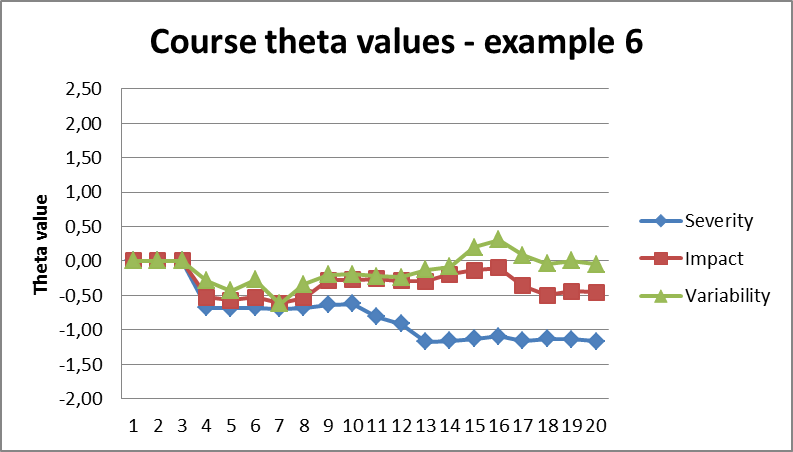


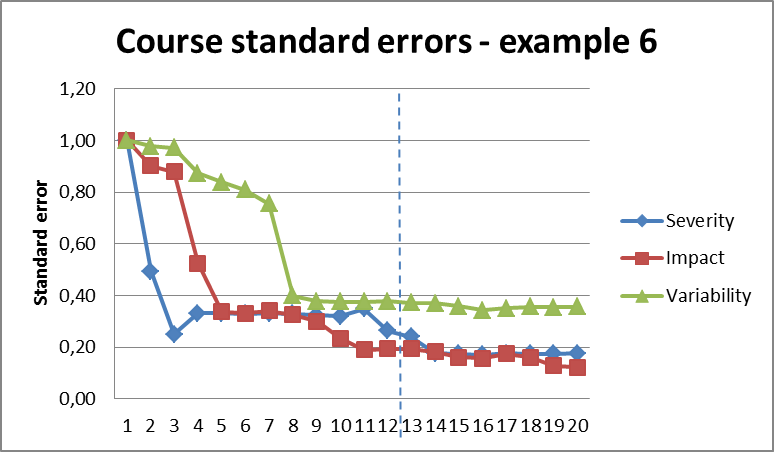


| item | | dimension | | | theta d1 | theta d2 | theta d3 | SE d1 | SE d2 | | SE d3 |
| --- | --- | --- | --- | --- | --- | --- | --- | --- | --- | --- | --- |
| 1 | | 1 | | | 0,0000 | 0,0000 | 0,0000 | 1,0000 | 1,0000 | | 1,0000 |
| 2 | | 1 | | | 0,0000 | 0,0000 | 0,0000 | 0,4939 | 0,9026 | | 0,9767 |
| 3 | | 2 | | | 0,0000 | 0,0000 | 0,0000 | 0,2494 | 0,8776 | | 0,9710 |
| 4 | | 2 | | | -0,6798 | -0,5298 | -0,2889 | 0,3307 | 0,5242 | | 0,8727 |
| 5 | | 3 | | | -0,6866 | -0,5726 | -0,4342 | 0,3305 | 0,3378 | | 0,8386 |
| 6 | | 3 | | | -0,6792 | -0,5254 | -0,2742 | 0,3294 | 0,3315 | | 0,8088 |
| 7 | | 3 | | | -0,6938 | -0,6169 | -0,6189 | 0,3316 | 0,3412 | | 0,7545 |
| 8 | | 3 | | | -0,6818 | -0,5364 | -0,3400 | 0,3298 | 0,3259 | | 0,3997 |
| 9 | | 2 | | | -0,6386 | -0,2828 | -0,2011 | 0,3232 | 0,2997 | | 0,3765 |
| 10 | | 2 | | | -0,6208 | -0,2696 | -0,1952 | 0,3203 | 0,2346 | | 0,3755 |
| 11 | | 1 | | | -0,8098 | -0,2569 | -0,2229 | 0,3493 | 0,1896 | | 0,3764 |
| 12 | | 1 | | | -0,9075 | -0,2897 | -0,2336 | 0,2632 | 0,1931 | | 0,3770 |
| 13 | | 1 | | | -1,1732 | -0,2926 | -0,1273 | 0,2404 | 0,1934 | | 0,3719 |
| 14 | | 2 | | | -1,1599 | -0,1957 | -0,0827 | 0,1757 | 0,1830 | | 0,3698 |
| 15 | | 3 | | | -1,1291 | -0,1383 | 0,1995 | 0,1734 | 0,1605 | | 0,3581 |
| 16 | | 2 | | | -1,0909 | -0,0977 | 0,3071 | 0,1707 | 0,1568 | | 0,3425 |
| 17 | | 2 | | | -1,1546 | -0,3578 | 0,0847 | 0,1753 | 0,1739 | | 0,3510 |
| 18 | | 2 | | | -1,1320 | -0,5007 | -0,0386 | 0,1736 | 0,1610 | | 0,3562 |
| 19 | | 2 | | | -1,1370 | -0,4382 | 0,0017 | 0,1740 | 0,1281 | | 0,3542 |
| 20 | | 1 | | | -1,1673 | -0,4558 | -0,0467 | 0,1763 | 0,1215 | | 0,3563 |
| item | dim. | | nr. | itemstam | | | | | | answer | |
| 1 | 1 | | 7 | I was particularly tired in the evening. | | | | | | 1-2 days | |
| 2 | 1 | | 10 | I was even tired if I hadn´t done anything special. | | | | | | rarely | |
| 3 | 2 | | 52 | Due to my fatigue I was not interested in anything. | | | | | | rarely | |
| 4 | 2 | | 17 | I felt weary. | | | | | | rarely | |
| 5 | 3 | | 187 | Exercise brings on my fatigue. | | | | | | no | |
| 6 | 3 | | 192 | Poor and / or little sleep is the cause of my fatigue. | | | | | | yes | |
| 7 | 3 | | 191 | Rheumatoid arthritis is the cause of my fatigue. | | | | | | I don’t know | |
| 8 | 3 | | 189 | Performance of routine daily activities increases my fatigue. | | | | | | yes | |
| 9 | 2 | | 138 | I really didn´t look forward to doing something due to the fatigue. | | | | | | a little | |
| 10 | 2 | | 38 | The fatigue felt like a physical burden. | | | | | | a little | |
| 11 | 1 | | 1 | Please circle the number that shows your average level of fatigue. | | | | | | 1 | |
| 12 | 1 | | 9 | How often have you been fatigued? | | | | | | occasionally but not most days | |
| 13 | 1 | | 8 | Did you feel fatigued? | | | | | | rarely | |
| 14 | 2 | | 134 | I had to make choices which things I would or would not do, because I was too tired to do everything. | | | | | | a little | |
| 15 | 3 | | 196 | My medication for rheumatism is the cause of my fatigue. | | | | | | yes | |
| 16 | 2 | | 150 | Fatigue meant I was less able to perform well. | | | | | | a little | |
| 17 | 2 | | 167 | Because of fatigue, I was less able to complete tasks that require physical effort. | | | | | | never | |
| 18 | 2 | | 33 | Physically I felt I was in a bad shape. | | | | | | rarely | |
| 19 | 2 | | 145 | Fatigue meant it took longer to finish things. | | | | | | a little | |
| 20 | 1 | | 11 | I soon tired whilst I was doing something. | | | | | | rarely | |

All items had a time frame of the last 7 days

Example 9


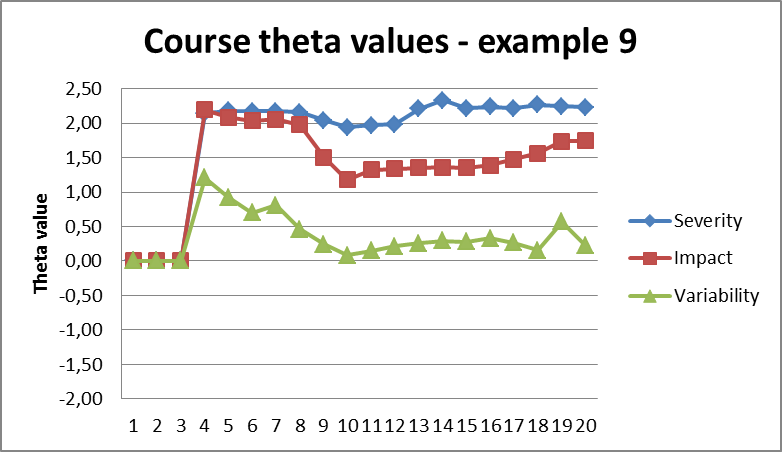


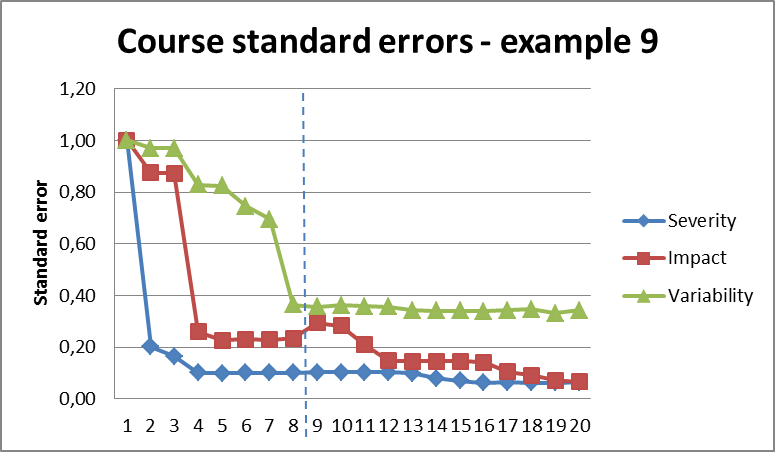


| item | | dimension | | theta d1 | theta d2 | theta d3 | SE d1 | SE d2 | SE d3 |
| --- | --- | --- | --- | --- | --- | --- | --- | --- | --- |
| 1 | | 1 | | 0,0000 | 0,0000 | 0,0000 | 1,0000 | 1,0000 | 1,0000 |
| 2 | | 1 | | 0,0000 | 0,0000 | 0,0000 | 0,2012 | 0,8746 | 0,9703 |
| 3 | | 2 | | 0,0000 | 0,0000 | 0,0000 | 0,1651 | 0,8727 | 0,9699 |
| 4 | | 2 | | 2,1412 | 2,1911 | 1,2110 | 0,1004 | 0,2594 | 0,8284 |
| 5 | | 3 | | 2,1805 | 2,0849 | 0,9213 | 0,0998 | 0,2264 | 0,8248 |
| 6 | | 3 | | 2,1725 | 2,0336 | 0,6962 | 0,0999 | 0,2311 | 0,7453 |
| 7 | | 3 | | 2,1741 | 2,0541 | 0,8056 | 0,0999 | 0,2286 | 0,6943 |
| 8 | | 3 | | 2,1607 | 1,9794 | 0,4580 | 0,1001 | 0,2346 | 0,3647 |
| 9 | | 2 | | 2,0445 | 1,5028 | 0,2451 | 0,1018 | 0,2939 | 0,3553 |
| 10 | | 2 | | 1,9394 | 1,1772 | 0,0804 | 0,1035 | 0,2828 | 0,3616 |
| 11 | | 2 | | 1,9684 | 1,3229 | 0,1495 | 0,1030 | 0,2087 | 0,3581 |
| 12 | | 3 | | 1,9814 | 1,3348 | 0,2134 | 0,1028 | 0,1475 | 0,3553 |
| 13 | | 1 | | 2,2093 | 1,3548 | 0,2590 | 0,0994 | 0,1455 | 0,3421 |
| 14 | | 1 | | 2,3289 | 1,3613 | 0,2925 | 0,0798 | 0,1449 | 0,3409 |
| 15 | | 1 | | 2,2172 | 1,3552 | 0,2765 | 0,0697 | 0,1455 | 0,3415 |
| 16 | | 2 | | 2,2346 | 1,3886 | 0,3317 | 0,0631 | 0,1422 | 0,3395 |
| 17 | | 2 | | 2,2183 | 1,4725 | 0,2641 | 0,0632 | 0,1050 | 0,3418 |
| 18 | | 2 | | 2,2651 | 1,5630 | 0,1556 | 0,0628 | 0,0906 | 0,3458 |
| 19 | | 2 | | 2,2439 | 1,7335 | 0,5700 | 0,0630 | 0,0721 | 0,3315 |
| 20 | | 3 | | 2,2322 | 1,7411 | 0,2253 | 0,0631 | 0,0665 | 0,3431 |
| item | dim. | nr. | itemstam | | | | | | answer |
| 1 | 1 | 5 | Did you feel tired? | | | | | | always |
| 2 | 1 | 10 | I was even tired if I hadn’t done anything special. | | | | | | always |
| 3 | 2 | 20 | I could sleep all day long. | | | | | | to a great extent |
| 4 | 2 | 43 | The fatigue made me dizzy. | | | | | | a little |
| 5 | 3 | 192 | Poor and / or little sleep is the cause of my fatigue. | | | | | | no |
| 6 | 3 | 190 | Stress brings on my fatigue. | | | | | | no |
| 7 | 3 | 191 | Rheumatoid arthritis is the cause of my fatigue. | | | | | | yes |
| 8 | 3 | 189 | Performance of routine daily activities increases my fatigue. | | | | | | no |
| 9 | 2 | 92 | Has being fatigued upset you? | | | | | | not at all |
| 10 | 2 | 157 | Fatigue made it difficult to conduct a (proper) converstation. | | | | | | not at all |
| 11 | 2 | 139 | Fatigue made it difficult to leave the house. | | | | | | rather |
| 12 | 3 | 196 | My medication for rheumatism is the cause of my fatigue. | | | | | | I don’t know |
| 13 | 1 | 3 | I felt fatigued. | | | | | | to a great extent |
| 14 | 1 | 2 | I felt tired. | | | | | | to a great extent |
| 15 | 1 | 11 | I soon tired whilst I was doing something. | | | | | | usually |
| 16 | 2 | 133 | Fatigue was a problem. | | | | | | rather |
| 17 | 2 | 84 | Fatigue has made me feel powerless. | | | | | | rather |
| 18 | 2 | 135 | Fatigue made it difficult to undertake anything new. | | | | | | to a great extent |
| 19 | 2 | 151 | To what degree has fatigue interfered with your ability to shop and do the errands? | | | | | | to a great extent |
| 20 | 3 | 186 | The severity of my fatigue could vary. | | | | | | no |

All items had a time frame of the last 7 days

Example 10


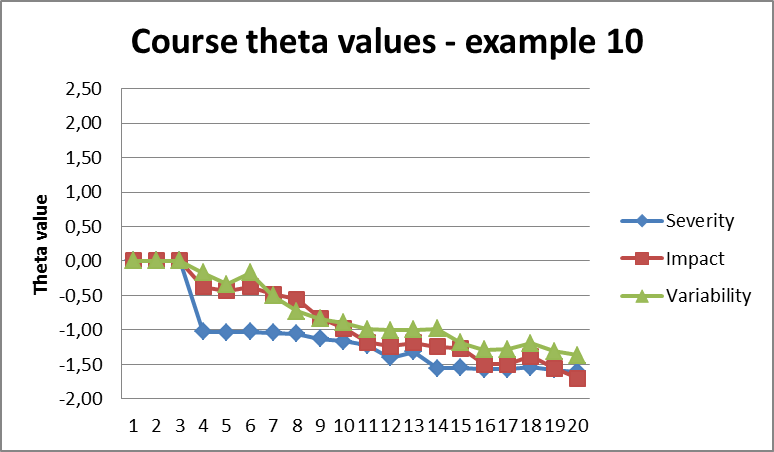


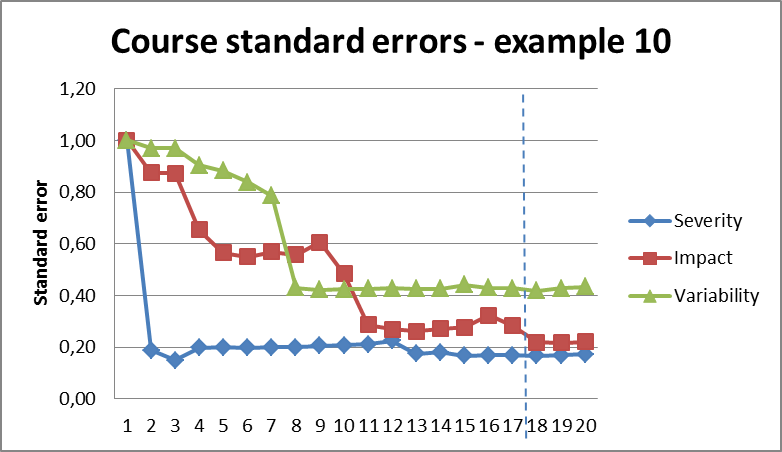


|  | item | | dimension | | | theta d1 | theta d2 | theta d3 | SE d1 | SE d2 | | SE d3 | |
| --- | --- | --- | --- | --- | --- | --- | --- | --- | --- | --- | --- | --- | --- |
|  | 1 | | 1 | | | 0,0000 | 0,0000 | 0,0000 | 1,0000 | 1,0000 | | 1,0000 | |
|  | 2 | | 1 | | | 0,0000 | 0,0000 | 0,0000 | 0,1877 | 0,8738 | | 0,9701 | |
|  | 3 | | 2 | | | 0,0000 | 0,0000 | 0,0000 | 0,1493 | 0,8720 | | 0,9697 | |
|  | 4 | | 2 | | | -1,0285 | -0,3748 | -0,1728 | 0,1983 | 0,6538 | | 0,9044 | |
|  | 5 | | 3 | | | -1,0362 | -0,4291 | -0,3391 | 0,1986 | 0,5660 | | 0,8825 | |
|  | 6 | | 3 | | | -1,0283 | -0,3735 | -0,1715 | 0,1981 | 0,5490 | | 0,8386 | |
|  | 7 | | 3 | | | -1,0427 | -0,4802 | -0,5014 | 0,1990 | 0,5685 | | 0,7851 | |
|  | 8 | | 3 | | | -1,0541 | -0,5583 | -0,7320 | 0,1997 | 0,5576 | | 0,4289 | |
|  | 9 | | 2 | | | -1,1264 | -0,8296 | -0,8360 | 0,2045 | 0,6052 | | 0,4225 | |
|  | 10 | | 2 | | | -1,1685 | -0,9777 | -0,8959 | 0,2071 | 0,4838 | | 0,4232 | |
|  | 11 | | 2 | | | -1,2207 | -1,1773 | -0,9893 | 0,2104 | 0,2862 | | 0,4257 | |
|  | 12 | | 1 | | | -1,4072 | -1,2372 | -1,0069 | 0,2255 | 0,2701 | | 0,4268 | |
|  | 13 | | 1 | | | -1,3152 | -1,1885 | -0,9970 | 0,1739 | 0,2612 | | 0,4259 | |
|  | 14 | | 1 | | | -1,5547 | -1,2419 | -0,9833 | 0,1804 | 0,2708 | | 0,4250 | |
|  | 15 | | 3 | | | -1,5472 | -1,2687 | -1,1878 | 0,1668 | 0,2759 | | 0,4417 | |
|  | 16 | | 2 | | | -1,5738 | -1,5002 | -1,2900 | 0,1690 | 0,3237 | | 0,4290 | |
|  | 17 | | 2 | | | -1,5704 | -1,5004 | -1,2805 | 0,1687 | 0,2824 | | 0,4274 | |
|  | 18 | | 2 | | | -1,5446 | -1,3807 | -1,1917 | 0,1666 | 0,2183 | | 0,4190 | |
|  | 19 | | 2 | | | -1,5770 | -1,5562 | -1,3071 | 0,1692 | 0,2162 | | 0,4286 | |
|  | 20 | | 2 | | | -1,6070 | -1,6999 | -1,3676 | 0,1717 | 0,2205 | | 0,4339 | |
| item | | dim. | | nr. | itemstam | | | | | | answer | |  |
| 1 | | 1 | | 8 | Did you feel fatigued? | | | | | | rarely | |  |
| 2 | | 1 | | 2 | I felt tired. | | | | | | a little | |  |
| 3 | | 2 | | 147 | My fatigue meant it was difficult to do voluntary work. | | | | | | a little | |  |
| 4 | | 2 | | 140 | Fatigue meant I had to force myself to get up in the morning and do something. | | | | | | not at all | |  |
| 5 | | 3 | | 185 | Could it vary how often you were fatigued? | | | | | | no | |  |
| 6 | | 3 | | 188 | Work brings on my fatigue. | | | | | | yes | |  |
| 7 | | 3 | | 191 | Rheumatoid arthritis is the cause of my fatigue. | | | | | | I don’t know | |  |
| 8 | | 3 | | 189 | Performance of routine daily activities increases my fatigue. | | | | | | no | |  |
| 9 | | 2 | | 138 | I really didn’t look forward to doing something due to the fatigue. | | | | | | not at all | |  |
| 10 | | 2 | | 33 | Physically I felt I was in a bad shape. | | | | | | rarely | |  |
| 11 | | 2 | | 145 | Fatigue meant it took longer to finish things. | | | | | | not at all | |  |
| 12 | | 1 | | 5 | Did you feel tired? | | | | | | rarely | |  |
| 13 | | 1 | | 3 | I felt fatigued. | | | | | | a little | |  |
| 14 | | 1 | | 11 | I soon tired whilst I was doing something. | | | | | | never | |  |
| 15 | | 3 | | 194 | Pain and / or other problems are the cause of my fatigue. | | | | | | no | |  |
| 16 | | 2 | | 164 | Fatigue prevented me from physically exerting myself for a longer period of time. | | | | | | never | |  |
| 17 | | 2 | | 109 | My fatigue made me less productive than I would have wished. | | | | | | rarely | |  |
| 18 | | 2 | | 137 | I was fatigued. | | | | | | a little | |  |
| 19 | | 2 | | 167 | Because of my fatigue, I was less able to complete tasks that require physical effort. | | | | | | never | |  |
| 20 | | 2 | | 127 | Because of my fatigue, I was less motivated to do anything that requires physical effort. | | | | | | never | |  |

All items had a time frame of the last 7 days

Example 12


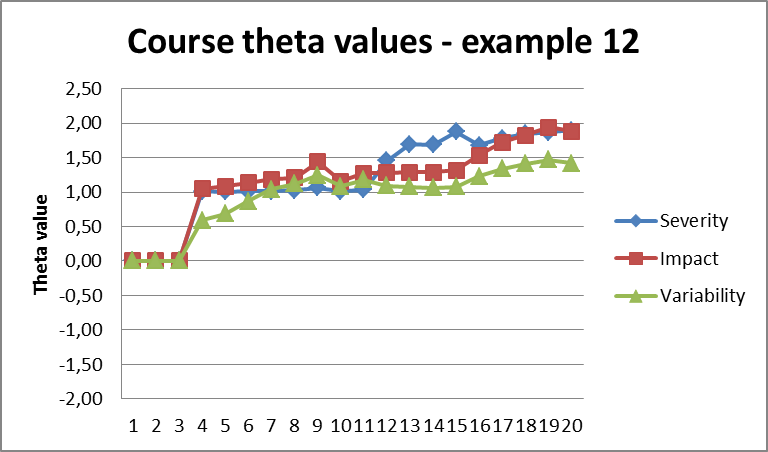


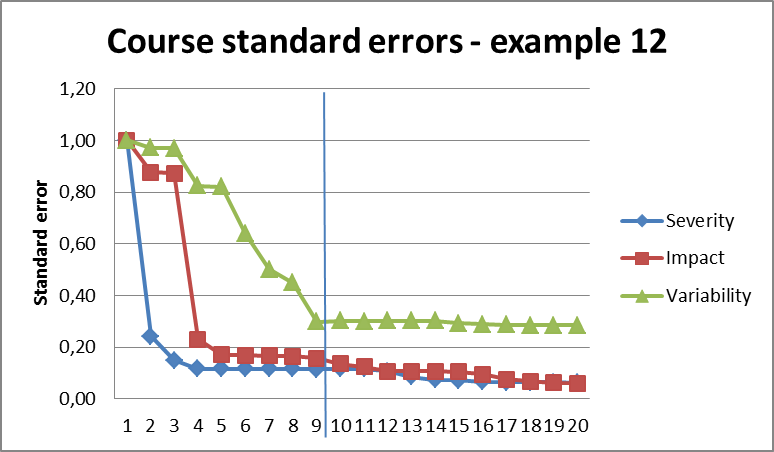


| item | | dimension | | | theta d1 | theta d2 | theta d3 | SE d1 | SE d2 | | SE d3 |  |
| --- | --- | --- | --- | --- | --- | --- | --- | --- | --- | --- | --- | --- |
| 1 | | 1 | | | 0,0000 | 0,0000 | 0,0000 | 1,0000 | 1,0000 | | 1,0000 |  |
| 2 | | 1 | | | 0,0000 | 0,0000 | 0,0000 | 0,2423 | 0,8771 | | 0,9709 |  |
| 3 | | 2 | | | 0,0000 | 0,0000 | 0,0000 | 0,1500 | 0,8721 | | 0,9697 |  |
| 4 | | 2 | | | 1,0087 | 1,0510 | 0,5897 | 0,1167 | 0,2307 | | 0,8253 |  |
| 5 | | 3 | | | 1,0035 | 1,0867 | 0,6817 | 0,1168 | 0,1710 | | 0,8199 |  |
| 6 | | 3 | | | 1,0110 | 1,1366 | 0,8690 | 0,1166 | 0,1684 | | 0,6411 |  |
| 7 | | 3 | | | 1,0140 | 1,1876 | 1,0431 | 0,1165 | 0,1660 | | 0,5022 |  |
| 8 | | 3 | | | 1,0267 | 1,2069 | 1,1182 | 0,1162 | 0,1651 | | 0,4490 |  |
| 9 | | 2 | | | 1,0589 | 1,4386 | 1,2441 | 0,1153 | 0,1563 | | 0,2987 |  |
| 10 | | 2 | | | 1,0103 | 1,1544 | 1,0875 | 0,1166 | 0,1362 | | 0,3017 |  |
| 11 | | 2 | | | 1,0319 | 1,2724 | 1,1766 | 0,1161 | 0,1231 | | 0,2999 |  |
| 12 | | 1 | | | 1,4516 | 1,2794 | 1,0935 | 0,1058 | 0,1072 | | 0,3015 |  |
| 13 | | 1 | | | 1,6929 | 1,2903 | 1,0738 | 0,0847 | 0,1067 | | 0,3019 |  |
| 14 | | 3 | | | 1,6878 | 1,2853 | 1,0607 | 0,0739 | 0,1069 | | 0,3021 |  |
| 15 | | 1 | | | 1,8723 | 1,3171 | 1,0786 | 0,0713 | 0,1054 | | 0,2921 |  |
| 16 | | 2 | | | 1,6788 | 1,5323 | 1,2294 | 0,0654 | 0,0957 | | 0,2890 |  |
| 17 | | 2 | | | 1,7833 | 1,7256 | 1,3350 | 0,0641 | 0,0761 | | 0,2869 |  |
| 18 | | 2 | | | 1,8455 | 1,8262 | 1,4081 | 0,0634 | 0,0682 | | 0,2855 |  |
| 19 | | 2 | | | 1,8664 | 1,9428 | 1,4681 | 0,0632 | 0,0630 | | 0,2845 |  |
| 20 | | 2 | | | 1,8962 | 1,8841 | 1,4168 | 0,0628 | 0,0601 | | 0,2854 |  |
| item | dim. | | nr. | itemstam | | | | | | answer | | |
| 1 | 1 | | 11 | I soon tired whilst I was doing something. | | | | | | usually | | |
| 2 | 1 | | 8 | Did you feel fatigued? | | | | | | usually | | |
| 3 | 2 | | 177 | Owing to my fatigue, I have taken to resting more often (e.g. sitting or lying down, a relaxing activity but not sleeping). | | | | | | usually | | |
| 4 | 2 | | 39 | I had less strength in my muscles because of my fatigue. | | | | | | rather | | |
| 5 | 3 | | 194 | Pain and / or other problems are the cause of my fatigue. | | | | | | yes | | |
| 6 | 3 | | 183 | To what degree has your fatigue changed? | | | | | | fatigue has gone up and down | | |
| 7 | 3 | | 189 | Performance of routine daily activities increases my fatigue. | | | | | | yes | | |
| 8 | 3 | | 191 | Rheumatoid arthritis is the cause of my fatigue. | | | | | | yes | | |
| 9 | 2 | | 139 | Fatigue made it difficult to leave the house. | | | | | | rather | | |
| 10 | 2 | | 157 | Fatigue made it difficult to conduct a (proper) conversation. | | | | | | not at all | | |
| 11 | 2 | | 149 | I was too tired to do my most important tasks. | | | | | | rather | | |
| 12 | 1 | | 3 | I felt fatigued. | | | | | | to a great extent | | |
| 13 | 1 | | 2 | I felt tired. | | | | | | to a great extent | | |
| 14 | 3 | | 196 | My medication for rheumatism is the cause of my fatigue. | | | | | | I don’t know | | |
| 15 | 1 | | 5 | Did you feel tired? | | | | | | always | | |
| 16 | 2 | | 133 | Fatigue was a problem. | | | | | | to a great extent | | |
| 17 | 2 | | 84 | Fatigue has made me feel powerless. | | | | | | not at all | | |
| 18 | 2 | | 92 | Has being fatigued upset you? | | | | | | rather | | |
| 19 | 2 | | 151 | To what degree has fatigue interfered with your ability to shop and do errands? | | | | | | to a great extent | | |
| 20 | 2 | | 86 | Have you felt down or depressed because of fatigue? | | | | | | a little | | |

All items had a time frame of the last 7 days
