# Supplementary material for: Working mechanism of a multidimensional computerized adaptive test for fatigue in rheumatoid arthritis
Source: Health Qual Life Outcomes. 2015 Feb 21;13:23. doi: 10.1186/s12955-015-0215-7 (PMC4340497; doi:10.1186/s12955-015-0215-7)
Supplement: Additional file 2: — Example 1, Example 2, Example 3, Example 4, Example 7, Example 8, Example 11, Example 13, Example 14, Example 15. For each of the examples, the following information is provided. Flowchart 1. Course of the theta scores per fatigue dimension over the administered items. Flowchart 2. Course of the standard errors per fatigue dimension over the administered items. Table S1. Theta scores and standard errors per dimension for each of the administered items. Table S2. Administered items (order, dimension, number in item bank, text and given answer). [file 12955_2015_215_MOESM2_ESM.doc]

Example 1


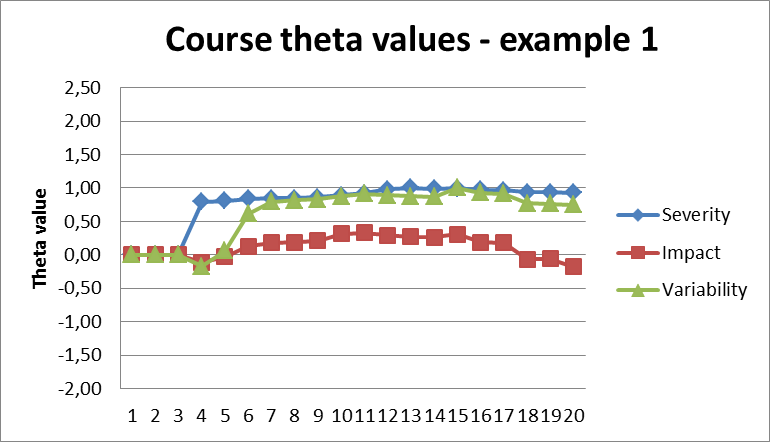


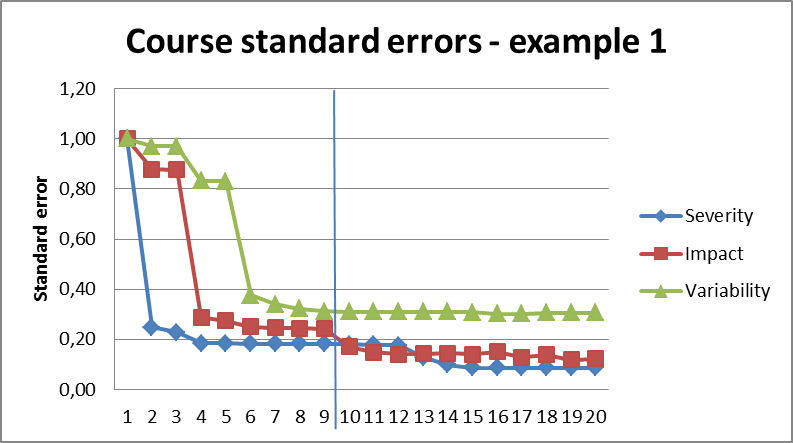


| item | | dimension | | | theta d1 | theta d2 | theta d3 | SE d1 | SE d2 | SE d3 |
| --- | --- | --- | --- | --- | --- | --- | --- | --- | --- | --- |
| 1 | | 1 | | | 0,0000 | 0,0000 | 0,0000 | 1,0000 | 1,0000 | 1,0000 |
| 2 | | 1 | | | 0,0000 | 0,0000 | 0,0000 | 0,2475 | 0,8775 | 0,9709 |
| 3 | | 2 | | | 0,0000 | 0,0000 | 0,0000 | 0,2286 | 0,8762 | 0,9707 |
| 4 | | 2 | | | 0,7953 | -0,1094 | -0,1684 | 0,1839 | 0,2874 | 0,8317 |
| 5 | | 3 | | | 0,8082 | -0,0316 | 0,0597 | 0,1834 | 0,2734 | 0,8300 |
| 6 | | 3 | | | 0,8346 | 0,1280 | 0,6146 | 0,1824 | 0,2510 | 0,3749 |
| 7 | | 3 | | | 0,8433 | 0,1814 | 0,7957 | 0,1821 | 0,2451 | 0,3390 |
| 8 | | 3 | | | 0,8445 | 0,1864 | 0,8159 | 0,1820 | 0,2445 | 0,3228 |
| 9 | | 2 | | | 0,8604 | 0,2107 | 0,8339 | 0,1814 | 0,2420 | 0,3116 |
| 10 | | 2 | | | 0,8912 | 0,3167 | 0,8770 | 0,1802 | 0,1707 | 0,3101 |
| 11 | | 2 | | | 0,9205 | 0,3293 | 0,9123 | 0,1792 | 0,1490 | 0,3091 |
| 12 | | 1 | | | 0,9792 | 0,2923 | 0,8887 | 0,1771 | 0,1409 | 0,3097 |
| 13 | | 1 | | | 1,0027 | 0,2724 | 0,8784 | 0,1290 | 0,1426 | 0,3100 |
| 14 | | 1 | | | 0,9843 | 0,2587 | 0,8659 | 0,0978 | 0,1437 | 0,3103 |
| 15 | | 3 | | | 0,9914 | 0,3070 | 1,0011 | 0,0857 | 0,1397 | 0,3070 |
| 16 | | 2 | | | 0,9712 | 0,1863 | 0,9251 | 0,0862 | 0,1501 | 0,3018 |
| 17 | | 2 | | | 0,9671 | 0,1822 | 0,9108 | 0,0862 | 0,1287 | 0,3021 |
| 18 | | 2 | | | 0,9354 | -0,0679 | 0,7731 | 0,0869 | 0,1386 | 0,3057 |
| 19 | | 2 | | | 0,9327 | -0,0597 | 0,7664 | 0,0870 | 0,1194 | 0,3058 |
| 20 | | 2 | | | 0,9255 | -0,1825 | 0,7445 | 0,0872 | 0,1220 | 0,3064 |
| item | dim. | | nr. | itemstam | | | | | | answer |
| 1 | 1 | | 1 | Please circle the number that shows your average level of fatigue. | | | | | | 4 |
| 2 | 1 | | 4 | Fatigue was the worst consequence of my rheumatism. | | | | | | 2 |
| 3 | 2 | | 101 | Fatigue meant I was unable to do as much as I would have liked. | | | | | | not at all |
| 4 | 2 | | 93 | Fatigue made me weepy. | | | | | | a little |
| 5 | 3 | | 191 | Rheumatoid arthritis is the cause of my fatigue. | | | | | | yes |
| 6 | 3 | | 183 | To what degree has your fatigue changed? | | | | | | increased |
| 7 | 3 | | 189 | Performance of routine daily activities increases my fatigue. | | | | | | yes |
| 8 | 3 | | 196 | My medication for rheumatism is the cause of my fatigue. | | | | | | I don’t know |
| 9 | 2 | | 138 | I really didn´t look forward to doing something due to the fatigue. | | | | | | a little |
| 10 | 2 | | 150 | Fatigue meant I was less able to perform well. | | | | | | a little |
| 11 | 2 | | 114 | I had to limit my social activities because I was tired. | | | | | | a little |
| 12 | 1 | | 3 | I felt fatigued. | | | | | | rather |
| 13 | 1 | | 8 | Did you feel fatigued? | | | | | | usually |
| 14 | 1 | | 2 | I felt tired. | | | | | | rather |
| 15 | 3 | | 186 | The severity of my fatigue could vary. | | | | | | yes |
| 16 | 2 | | 38 | The fatigue felt like a physical burden. | | | | | | a little |
| 17 | 2 | | 134 | I had to make choices which things I would or would not do, because I was too tired to do everything. | | | | | | a little |
| 18 | 2 | | 167 | Because of fatigue, I was less able to complete tasks that require physical effort. | | | | | | never |
| 19 | 2 | | 104 | Fatigue meant that everything I did was such an effort. | | | | | | a little |
| 20 | 2 | | 35 | I felt weak due to my fatigue. | | | | | | not at all |

All items had a time frame of the last 7 days

Example 2


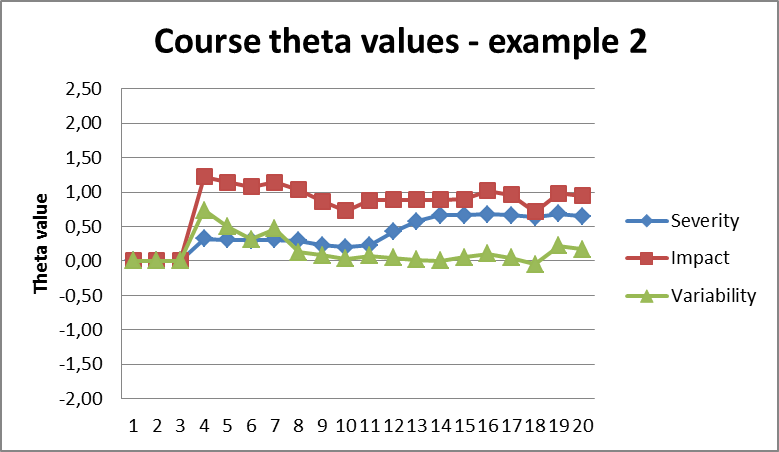


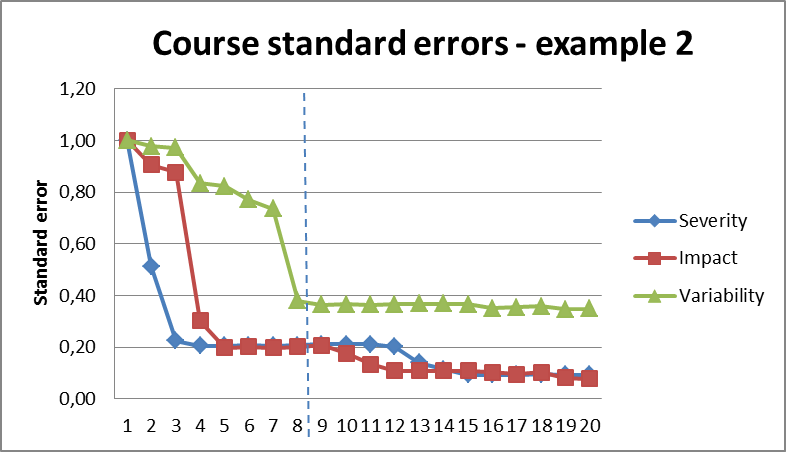


| item | dimension | | theta d1 | | theta d2 | theta d3 | SE d1 | SE d2 | SE d3 |
| --- | --- | --- | --- | --- | --- | --- | --- | --- | --- |
| 1 | 1 | | 0,0000 | | 0,0000 | 0,0000 | 1,0000 | 1,0000 | 1,0000 |
| 2 | 1 | | 0,0000 | | 0,0000 | 0,0000 | 0,5121 | 0,9051 | 0,9772 |
| 3 | 2 | | 0,0000 | | 0,0000 | 0,0000 | 0,2245 | 0,8760 | 0,9706 |
| 4 | 2 | | 0,3251 | | 1,2201 | 0,7356 | 0,2055 | 0,3019 | 0,8336 |
| 5 | 3 | | 0,3101 | | 1,1392 | 0,4948 | 0,2062 | 0,1985 | 0,8222 |
| 6 | 3 | | 0,2987 | | 1,0775 | 0,3122 | 0,2068 | 0,2006 | 0,7701 |
| 7 | 3 | | 0,3110 | | 1,1442 | 0,4697 | 0,2061 | 0,1979 | 0,7361 |
| 8 | 3 | | 0,2899 | | 1,0300 | 0,1288 | 0,2072 | 0,2009 | 0,3797 |
| 9 | 2 | | 0,2306 | | 0,8700 | 0,0849 | 0,2102 | 0,2071 | 0,3646 |
| 10 | 2 | | 0,2010 | | 0,7288 | 0,0305 | 0,2118 | 0,1767 | 0,3666 |
| 11 | 2 | | 0,2277 | | 0,8817 | 0,0792 | 0,2103 | 0,1330 | 0,3643 |
| 12 | 1 | | 0,4222 | | 0,8861 | 0,0435 | 0,2006 | 0,1081 | 0,3657 |
| 13 | 1 | | 0,5778 | | 0,8890 | 0,0149 | 0,1396 | 0,1079 | 0,3668 |
| 14 | 1 | | 0,6669 | | 0,8908 | 0,0011 | 0,1145 | 0,1078 | 0,3674 |
| 15 | 3 | | 0,6632 | | 0,8931 | 0,0520 | 0,0935 | 0,1077 | 0,3653 |
| 16 | 2 | | 0,6766 | | 1,0207 | 0,1074 | 0,0932 | 0,1019 | 0,3513 |
| 17 | 2 | | 0,6597 | | 0,9622 | 0,0487 | 0,0936 | 0,0956 | 0,3537 |
| 18 | 2 | | 0,6375 | | 0,7157 | -0,0458 | 0,0942 | 0,1018 | 0,3578 |
| 19 | 2 | | 0,6837 | | 0,9860 | 0,2210 | 0,0930 | 0,0819 | 0,3469 |
| 20 | 3 | | 0,6504 | | 0,9464 | 0,1693 | 0,0939 | 0,0769 | 0,3488 |
| item | dim. | nr. | | itemstam | | | | | answer |
| 1 | 1 | 4 | | Fatigue was the worst consequence of my rheumatism. | | | | | 3 |
| 2 | 1 | 11 | | I soon tired whilst I was doing something. | | | | | sometimes |
| 3 | 2 | 46 | | I had energy. | | | | | not at all |
| 4 | 2 | 62 | | How often did you feel less alert owing to your fatigue? | | | | | often |
| 5 | 3 | 184 | | The duration of my fatigue could vary. | | | | | no |
| 6 | 3 | 185 | | Could it vary how often you were fatigued? | | | | | no |
| 7 | 3 | 191 | | Rheumatoid arthritis is the cause of my fatigue. | | | | | yes |
| 8 | 3 | 189 | | Performance of routine daily activities increases my fatigue. | | | | | no |
| 9 | 2 | 149 | | I was too tired to do my most important tasks. | | | | | a little |
| 10 | 2 | 150 | | Fatigue meant I was less able to perform well. | | | | | a little |
| 11 | 2 | 138 | | I really didn´t look forward to doing something due to the fatigue. | | | | | rather |
| 12 | 1 | 2 | | I felt tired. | | | | | rather |
| 13 | 1 | 3 | | I felt fatigued. | | | | | rather |
| 14 | 1 | 8 | | Did you feel fatigued? | | | | | usually |
| 15 | 3 | 196 | | My medication for rheumatism is the cause of my fatigue. | | | | | I don’t know |
| 16 | 2 | 114 | | I had to limit my social activities because I was tired. | | | | | rather |
| 17 | 2 | 139 | | Fatigue made it difficult to leave the house. | | | | | a little |
| 18 | 2 | 135 | | Fatigue made it difficult to undertake anything new. | | | | | not at all |
| 19 | 2 | 134 | | I had to make choices which things I would or would not do, because I was too tired to do everything. | | | | | to a great extent |
| 20 | 3 | 186 | | The severity of my fatigue could vary. | | | | | yes |

All items had a time frame of the last 7 days

Example 3


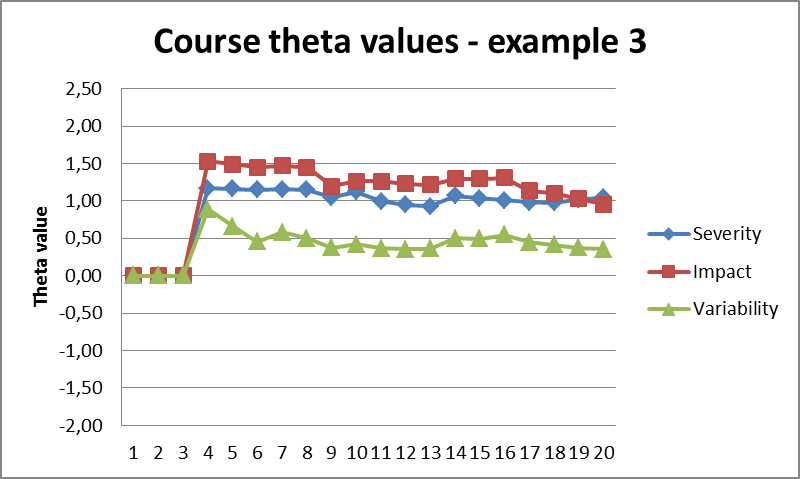


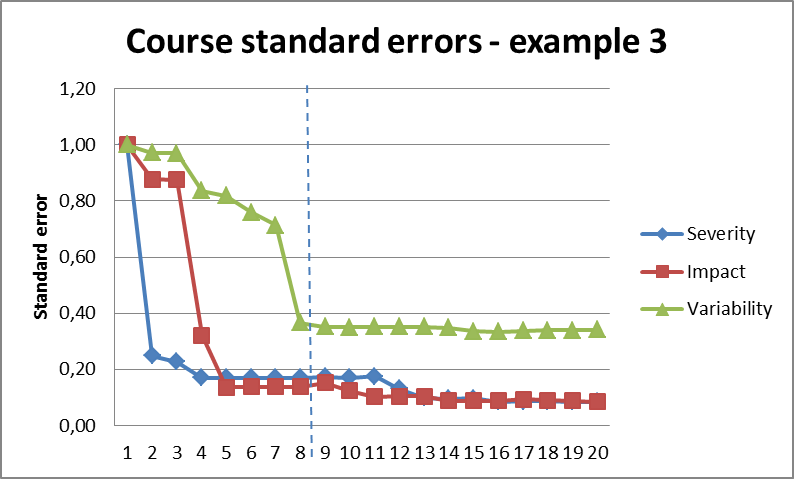


| item | | dimension | | | theta d1 | theta d2 | theta d3 | SE d1 | SE d2 | SE d3 |
| --- | --- | --- | --- | --- | --- | --- | --- | --- | --- | --- |
| 1 | | 1 | | | 0,0000 | 0,0000 | 0,0000 | 1,0000 | 1,0000 | 1,0000 |
| 2 | | 1 | | | 0,0000 | 0,0000 | 0,0000 | 0,2475 | 0,8775 | 0,9709 |
| 3 | | 2 | | | 0,0000 | 0,0000 | 0,0000 | 0,2269 | 0,8761 | 0,9706 |
| 4 | | 2 | | | 1,1678 | 1,5321 | 0,8870 | 0,1692 | 0,3208 | 0,8362 |
| 5 | | 3 | | | 1,1595 | 1,4896 | 0,6609 | 0,1694 | 0,1363 | 0,8175 |
| 6 | | 3 | | | 1,1521 | 1,4498 | 0,4549 | 0,1696 | 0,1382 | 0,7597 |
| 7 | | 3 | | | 1,1541 | 1,4698 | 0,5808 | 0,1695 | 0,1371 | 0,7135 |
| 8 | | 3 | | | 1,1523 | 1,4506 | 0,5040 | 0,1696 | 0,1377 | 0,3651 |
| 9 | | 2 | | | 1,0519 | 1,1987 | 0,3723 | 0,1727 | 0,1513 | 0,3519 |
| 10 | | 2 | | | 1,1222 | 1,2593 | 0,4212 | 0,1705 | 0,1228 | 0,3501 |
| 11 | | 1 | | | 0,9941 | 1,2644 | 0,3660 | 0,1746 | 0,1015 | 0,3519 |
| 12 | | 1 | | | 0,9495 | 1,2319 | 0,3539 | 0,1299 | 0,1031 | 0,3523 |
| 13 | | 2 | | | 0,9293 | 1,2159 | 0,3583 | 0,0988 | 0,1039 | 0,3521 |
| 14 | | 3 | | | 1,0713 | 1,2928 | 0,4988 | 0,0954 | 0,0879 | 0,3476 |
| 15 | | 1 | | | 1,0332 | 1,2984 | 0,4965 | 0,0963 | 0,0876 | 0,3357 |
| 16 | | 2 | | | 1,0111 | 1,3081 | 0,5459 | 0,0851 | 0,0873 | 0,3342 |
| 17 | | 2 | | | 0,9815 | 1,1359 | 0,4502 | 0,0857 | 0,0925 | 0,3372 |
| 18 | | 2 | | | 0,9769 | 1,0994 | 0,4128 | 0,0858 | 0,0897 | 0,3385 |
| 19 | | 2 | | | 1,0207 | 1,0278 | 0,3766 | 0,0849 | 0,0876 | 0,3397 |
| 20 | | 2 | | | 1,0417 | 0,9556 | 0,3559 | 0,0844 | 0,0837 | 0,3404 |
| item | dim. | | nr. | itemstam | | | | | | answer |
| 1 | 1 | | 1 | Please circle the number that shows your average level of fatigue. | | | | | | 3 |
| 2 | 1 | | 7 | I was particularly tired in the evening. | | | | | | every day |
| 3 | 2 | | 161 | Has fatigue made it difficult to bath or shower? | | | | | | a little |
| 4 | 2 | | 137 | I was fatigued. | | | | | | to a great extent |
| 5 | 3 | | 190 | Stress brings on my fatigue. | | | | | | no |
| 6 | 3 | | 184 | The duration of my fatigue could vary. | | | | | | no |
| 7 | 3 | | 191 | Rheumatoid arthritis is the cause of my fatigue. | | | | | | yes |
| 8 | 3 | | 189 | Performance of routine daily activities increases my fatigue. | | | | | | I don’t know |
| 9 | 2 | | 139 | Fatigue made it difficult to leave the house. | | | | | | a little |
| 10 | 2 | | 135 | Fatigue made it difficult to undertake anything new. | | | | | | rather |
| 11 | 1 | | 3 | I felt fatigued. | | | | | | rather |
| 12 | 1 | | 8 | Did you feel fatigued? | | | | | | usually |
| 13 | 2 | | 133 | Fatigue was a problem. | | | | | | rather |
| 14 | 3 | | 196 | My medication for rheumatism is the cause of my fatigue. | | | | | | I don’t know |
| 15 | 1 | | 2 | I felt tired. | | | | | | rahter |
| 16 | 2 | | 157 | Fatigue made it difficult to conduct a (proper) conversation. | | | | | | a little |
| 17 | 2 | | 84 | Fatigue has made me feel powerless. | | | | | | not at all |
| 18 | 2 | | 149 | I was too tired to do my most important tasks. | | | | | | a little |
| 19 | 2 | | 150 | Fatigue meant I was less able to perform well. | | | | | | a little |
| 20 | 2 | | 105 | Has fatigue made it difficult to do your work or other daily activities? | | | | | | a little |

All items had a time frame of the last 7 days

Example 4


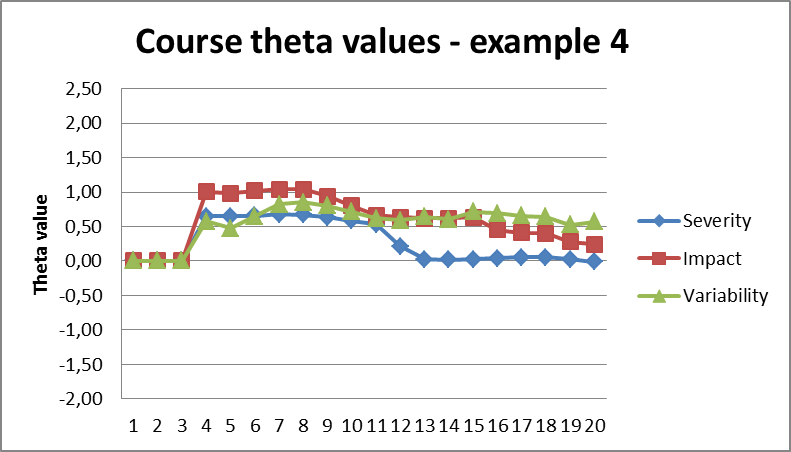


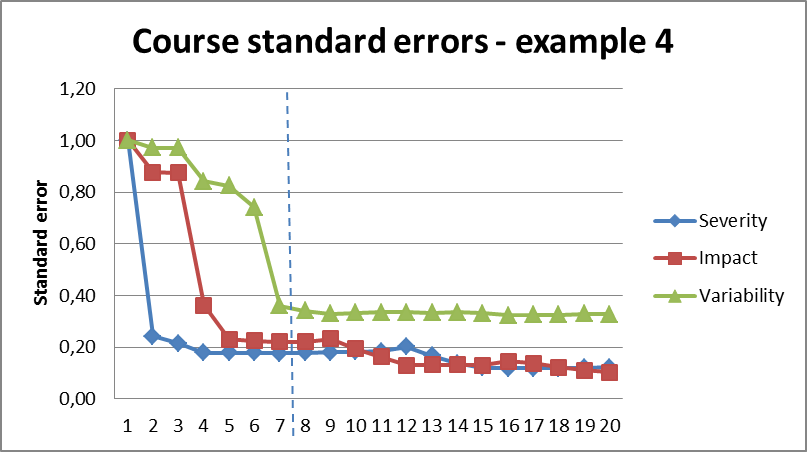


| item | dimension | | | theta d1 | theta d2 | theta d3 | SE d1 | SE d2 | SE d3 |
| --- | --- | --- | --- | --- | --- | --- | --- | --- | --- |
| 1 | 1 | | | 0,0000 | 0,0000 | 0,0000 | 1,0000 | 1,0000 | 1,0000 |
| 2 | 1 | | | 0,0000 | 0,0000 | 0,0000 | 0,2423 | 0,8771 | 0,9709 |
| 3 | 2 | | | 0,0000 | 0,0000 | 0,0000 | 0,2145 | 0,8754 | 0,9705 |
| 4 | 2 | | | 0,6509 | 1,0039 | 0,5751 | 0,1785 | 0,3615 | 0,8423 |
| 5 | 3 | | | 0,6475 | 0,9823 | 0,4740 | 0,1785 | 0,2299 | 0,8252 |
| 6 | 3 | | | 0,6527 | 1,0186 | 0,6455 | 0,1783 | 0,2245 | 0,7409 |
| 7 | 3 | | | 0,6769 | 1,0374 | 0,8226 | 0,1771 | 0,2202 | 0,3610 |
| 8 | 3 | | | 0,6706 | 1,0370 | 0,8507 | 0,1774 | 0,2202 | 0,3414 |
| 9 | 2 | | | 0,6324 | 0,9402 | 0,8009 | 0,1793 | 0,2327 | 0,3300 |
| 10 | 2 | | | 0,5863 | 0,8004 | 0,7139 | 0,1815 | 0,1934 | 0,3320 |
| 11 | 2 | | | 0,5362 | 0,6620 | 0,6178 | 0,1840 | 0,1620 | 0,3346 |
| 12 | 1 | | | 0,2145 | 0,6329 | 0,5940 | 0,2009 | 0,1302 | 0,3351 |
| 13 | 1 | | | 0,0267 | 0,6222 | 0,6441 | 0,1664 | 0,1310 | 0,3337 |
| 14 | 1 | | | 0,0161 | 0,6226 | 0,6064 | 0,1380 | 0,1310 | 0,3347 |
| 15 | 3 | | | 0,0247 | 0,6348 | 0,7166 | 0,1198 | 0,1300 | 0,3317 |
| 16 | 2 | | | 0,0415 | 0,4520 | 0,6923 | 0,1191 | 0,1461 | 0,3243 |
| 17 | 2 | | | 0,0542 | 0,4109 | 0,6540 | 0,1186 | 0,1374 | 0,3253 |
| 18 | 2 | | | 0,0515 | 0,4056 | 0,6420 | 0,1187 | 0,1223 | 0,3256 |
| 19 | 2 | | | 0,0245 | 0,2816 | 0,5224 | 0,1198 | 0,1114 | 0,3292 |
| 20 | 2 | | | -0,0123 | 0,2413 | 0,5661 | 0,1213 | 0,1032 | 0,3278 |
| item | dim. | nr. | itemstam | | | | | | answer |
| 1 | 1 | 11 | I soon tired whilst I was doing something. | | | | | | usually |
| 2 | 1 | 13 | How many hours per day, on average, were you fatigued? | | | | | | appr. 1-4 hours |
| 3 | 2 | 80 | Have you felt you have less control about your life because of fatigue? | | | | | | a little |
| 4 | 2 | 170 | Have you cancelled plans because of fatigue? e.g. plans to go out, or do jobs around the home or garden. | | | | | | rather |
| 5 | 3 | 193 | Too much going on / too many activities are the cause of my fatigue. | | | | | | I don’t know |
| 6 | 3 | 191 | Rheumatoid arthritis is the cause of my fatigue. | | | | | | yes |
| 7 | 3 | 189 | Performance of routine daily activities increases my fatigue. | | | | | | yes |
| 8 | 3 | 196 | My medication for rheumatism is the cause of my fatigue. | | | | | | I don’t know |
| 9 | 2 | 149 | I was too tired to do my most important tasks. | | | | | | a little |
| 10 | 2 | 150 | Fatigue meant I was less able to perform well. | | | | | | a little |
| 11 | 2 | 138 | I really didn´t look forward to doing something due to the fatigue. | | | | | | a little |
| 12 | 1 | 3 | I felt fatigued. | | | | | | a little |
| 13 | 1 | 2 | I felt tired. | | | | | | a little |
| 14 | 1 | 9 | How often have you been fatigued? | | | | | | most, but not all days |
| 15 | 3 | 186 | The severity of my fatigue could vary. | | | | | | yes |
| 16 | 2 | 114 | I had to limit my social activities because I was tired. | | | | | | not at all |
| 17 | 2 | 134 | I had to make choices which things I would or would not do, because I was too tired to do everything. | | | | | | a little |
| 18 | 2 | 167 | Because of my fatigue, I was less able to complete tasks that require physical effort. | | | | | | sometimes |
| 19 | 2 | 38 | The fatigue felt like a physical burden. | | | | | | a little |
| 20 | 2 | 139 | Fatigue made it difficult to leave the house. | | | | | | not at all |

All items had a time frame of the last 7 days

Example 7


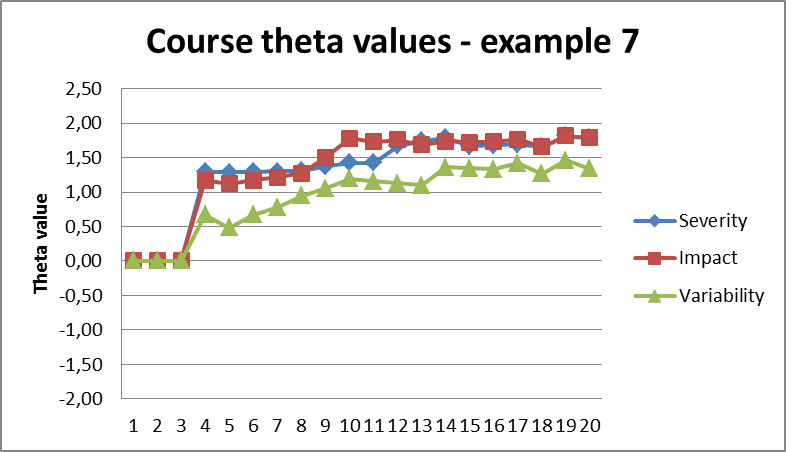


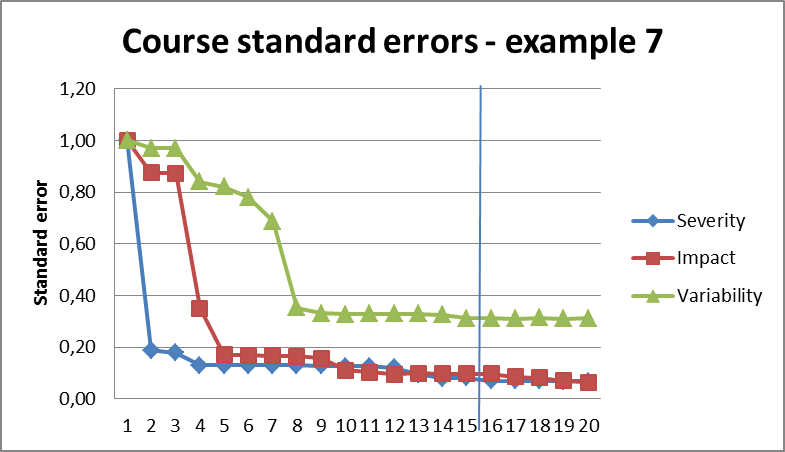


| item | dimension | | | theta d1 | theta d2 | theta d3 | SE d1 | SE d2 | | SE d3 |  |
| --- | --- | --- | --- | --- | --- | --- | --- | --- | --- | --- | --- |
| 1 | 1 | | | 0,0000 | 0,0000 | 0,0000 | 1,0000 | 1,0000 | | 1,0000 |  |
| 2 | 1 | | | 0,0000 | 0,0000 | 0,0000 | 0,1877 | 0,8738 | | 0,9701 |  |
| 3 | 2 | | | 0,0000 | 0,0000 | 0,0000 | 0,1777 | 0,8733 | | 0,9700 |  |
| 4 | 2 | | | 1,2963 | 1,1735 | 0,6736 | 0,1300 | 0,3512 | | 0,8407 |  |
| 5 | 3 | | | 1,2882 | 1,1179 | 0,4828 | 0,1302 | 0,1708 | | 0,8199 |  |
| 6 | 3 | | | 1,2961 | 1,1747 | 0,6691 | 0,1299 | 0,1686 | | 0,7794 |  |
| 7 | 3 | | | 1,3021 | 1,2180 | 0,7815 | 0,1298 | 0,1668 | | 0,6862 |  |
| 8 | 3 | | | 1,3102 | 1,2710 | 0,9489 | 0,1295 | 0,1643 | | 0,3514 |  |
| 9 | 2 | | | 1,3745 | 1,5019 | 1,0547 | 0,1278 | 0,1571 | | 0,3308 |  |
| 10 | 2 | | | 1,4272 | 1,7781 | 1,1939 | 0,1264 | 0,1110 | | 0,3278 |  |
| 11 | 2 | | | 1,4280 | 1,7305 | 1,1545 | 0,1264 | 0,1029 | | 0,3286 |  |
| 12 | 1 | | | 1,6738 | 1,7599 | 1,1290 | 0,1207 | 0,0959 | | 0,3290 |  |
| 13 | 1 | | | 1,7409 | 1,6849 | 1,0982 | 0,0943 | 0,0991 | | 0,3297 |  |
| 14 | 3 | | | 1,7881 | 1,7366 | 1,3591 | 0,0789 | 0,0969 | | 0,3247 |  |
| 15 | 1 | | | 1,6663 | 1,7235 | 1,3462 | 0,0809 | 0,0975 | | 0,3120 |  |
| 16 | 2 | | | 1,6796 | 1,7305 | 1,3289 | 0,0699 | 0,0972 | | 0,3123 |  |
| 17 | 2 | | | 1,6903 | 1,7648 | 1,4150 | 0,0697 | 0,0857 | | 0,3106 |  |
| 18 | 2 | | | 1,6567 | 1,6562 | 1,2654 | 0,0702 | 0,0809 | | 0,3135 |  |
| 19 | 2 | | | 1,8182 | 1,8145 | 1,4597 | 0,0680 | 0,0707 | | 0,3098 |  |
| 20 | 3 | | | 1,7879 | 1,7888 | 1,3403 | 0,0684 | 0,0644 | | 0,3120 |  |
| item | dim. | nr. | itemstam | | | | | | answer | | |
| 1 | 1 | 8 | Did you feel fatigued? | | | | | | always | | |
| 2 | 1 | 12 | How long, on average, has each episode of fatigue lasted? | | | | | | several hours | | |
| 3 | 2 | 173 | I would describe my fatigue as normal / abnormal. | | | | | | 4 | | |
| 4 | 2 | 48 | When I did something I was quickly exhausted. | | | | | | usually | | |
| 5 | 3 | 185 | Could it vary how often you were fatigued? | | | | | | no | | |
| 6 | 3 | 186 | The severity of my fatigue could vary. | | | | | | yes | | |
| 7 | 3 | 191 | Rheumatoid arthritis is the cause of my fatigue. | | | | | | yes | | |
| 8 | 3 | 189 | Performance of routine daily activities increases my fatigue. | | | | | | yes | | |
| 9 | 2 | 139 | Fatigue made it difficult to leave the house. | | | | | | rather | | |
| 10 | 2 | 157 | Fatigue made it difficult to conduct a (proper) conversation. | | | | | | rather | | |
| 11 | 2 | 92 | Has being fatigued upset you? | | | | | | a little | | |
| 12 | 1 | 3 | I felt fatigued. | | | | | | to a great extent | | |
| 13 | 1 | 2 | I felt tired. | | | | | | to a great extent | | |
| 14 | 3 | 196 | My medication for rheumatism is the cause of my fatigue. | | | | | | yes | | |
| 15 | 1 | 5 | Did you feel tired? | | | | | | usually | | |
| 16 | 2 | 84 | Fatigue has made me feel powerless. | | | | | | rather | | |
| 17 | 2 | 151 | To what degree has fatigue interfered with your ability to shop and do errands? | | | | | | rather | | |
| 18 | 2 | 118 | Fatigue restricted me in my contact with family, friends and acquaintances. | | | | | | a little | | |
| 19 | 2 | 133 | Fatigue was a problem. | | | | | | to a great extent | | |
| 20 | 3 | 183 | To what degree has your fatigue changed? | | | | | | stayed the same | | |

All items had a time frame of the last 7 days

Example 8


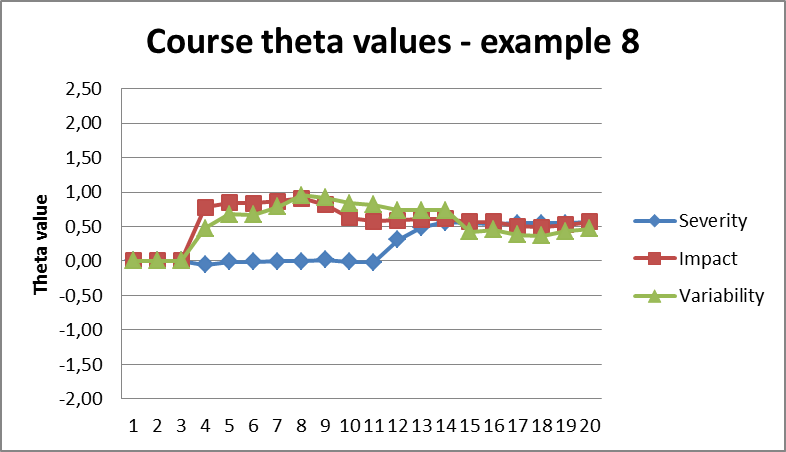


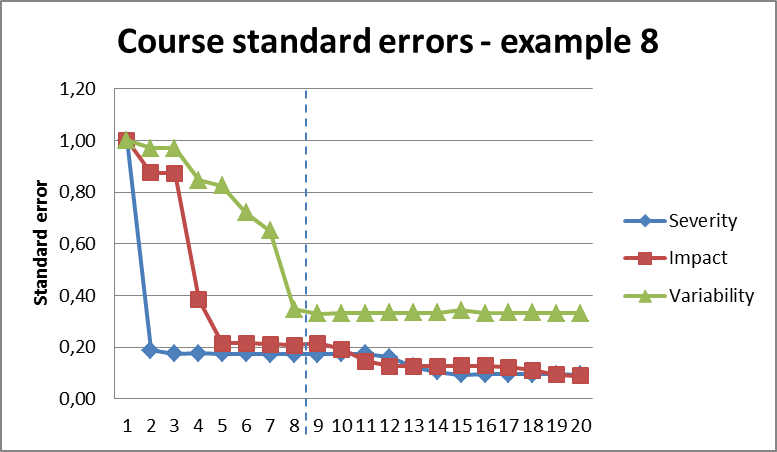


|  | item | | dimension | | | theta d1 | theta d2 | theta d3 | SE d1 | SE d2 | | SE d3 | |
| --- | --- | --- | --- | --- | --- | --- | --- | --- | --- | --- | --- | --- | --- |
|  | 1 | | 1 | | | 0,0000 | 0,0000 | 0,0000 | 1,0000 | 1,0000 | | 1,0000 | |
|  | 2 | | 1 | | | 0,0000 | 0,0000 | 0,0000 | 0,1877 | 0,8738 | | 0,9701 | |
|  | 3 | | 2 | | | 0,0000 | 0,0000 | 0,0000 | 0,1738 | 0,8731 | | 0,9700 | |
|  | 4 | | 2 | | | -0,0563 | 0,7782 | 0,4735 | 0,1756 | 0,3835 | | 0,8458 | |
|  | 5 | | 3 | | | -0,0143 | 0,8419 | 0,6785 | 0,1737 | 0,2152 | | 0,8237 | |
|  | 6 | | 3 | | | -0,0146 | 0,8399 | 0,6708 | 0,1737 | 0,2147 | | 0,7198 | |
|  | 7 | | 3 | | | -0,0069 | 0,8664 | 0,7867 | 0,1734 | 0,2121 | | 0,6509 | |
|  | 8 | | 3 | | | -0,0009 | 0,9068 | 0,9511 | 0,1731 | 0,2075 | | 0,3461 | |
|  | 9 | | 2 | | | 0,0165 | 0,8135 | 0,9179 | 0,1723 | 0,2153 | | 0,3295 | |
|  | 10 | | 2 | | | -0,0121 | 0,6294 | 0,8379 | 0,1736 | 0,1918 | | 0,3313 | |
|  | 11 | | 2 | | | -0,0168 | 0,5782 | 0,8147 | 0,1737 | 0,1461 | | 0,3316 | |
|  | 12 | | 1 | | | 0,3068 | 0,5905 | 0,7391 | 0,1599 | 0,1269 | | 0,3335 | |
|  | 13 | | 1 | | | 0,4863 | 0,6025 | 0,7343 | 0,1232 | 0,1260 | | 0,3337 | |
|  | 14 | | 1 | | | 0,5650 | 0,6089 | 0,7336 | 0,1057 | 0,1256 | | 0,3337 | |
|  | 15 | | 3 | | | 0,5538 | 0,5682 | 0,4272 | 0,0940 | 0,1285 | | 0,3428 | |
|  | 16 | | 2 | | | 0,5397 | 0,5684 | 0,4552 | 0,0944 | 0,1285 | | 0,3307 | |
|  | 17 | | 2 | | | 0,5493 | 0,5068 | 0,3773 | 0,0941 | 0,1227 | | 0,3333 | |
|  | 18 | | 2 | | | 0,5465 | 0,4909 | 0,3645 | 0,0942 | 0,1112 | | 0,3337 | |
|  | 19 | | 2 | | | 0,5455 | 0,5228 | 0,4314 | 0,0942 | 0,0938 | | 0,3314 | |
|  | 20 | | 2 | | | 0,5646 | 0,5693 | 0,4661 | 0,0937 | 0,0885 | | 0,3302 | |
| item | | dim. | | nr. | itemstam | | | | | | answer | |  |
| 1 | | 1 | | 8 | Did you feel fatigued? | | | | | | sometimes | |  |
| 2 | | 1 | | 13 | How many hours per day, on average, were you fatigued? | | | | | | appr. 1-4 hours | |  |
| 3 | | 2 | | 173 | I would describe my fatigue as normal / abnormal. | | | | | | 3 | |  |
| 4 | | 2 | | 75 | When I was tired, everything was too much (hassle). | | | | | | rather | |  |
| 5 | | 3 | | 186 | The severity of my fatigue could vary. | | | | | | yes | |  |
| 6 | | 3 | | 195 | One of the reasons for my fatigue is caused by the medication I have to take. | | | | | | I don’t know | |  |
| 7 | | 3 | | 191 | Rheumatoid arthritis is the cause of my fatigue. | | | | | | yes | |  |
| 8 | | 3 | | 189 | Performance of routine daily activities increases my fatigue. | | | | | | yes | |  |
| 9 | | 2 | | 149 | I was too tired to do my most important tasks. | | | | | | a little | |  |
| 10 | | 2 | | 138 | I really didn´t look forward to doing something due to the fatigue. | | | | | | a little | |  |
| 11 | | 2 | | 150 | Fatigue meant I was less able to perform well. | | | | | | a little | |  |
| 12 | | 1 | | 9 | How often have you been fatigued? | | | | | | each day | |  |
| 13 | | 1 | | 3 | I felt fatigued. | | | | | | rather | |  |
| 14 | | 1 | | 2 | I felt tired. | | | | | | rather | |  |
| 15 | | 3 | | 196 | My medication for rheumatism is the cause of my fatigue. | | | | | | no | |  |
| 16 | | 2 | | 114 | I had to limit my social activities because I was tired. | | | | | | a little | |  |
| 17 | | 2 | | 134 | I had to make choices which things I would or would not do, because I was too tired to do everything. | | | | | | a little | |  |
| 18 | | 2 | | 167 | Because of fatigue, I was less able to complete tasks that require physical effort. | | | | | | sometimes | |  |
| 19 | | 2 | | 139 | Fatigue made it difficult to leave the house? | | | | | | a little | |  |
| 20 | | 2 | | 146 | Fatigue meant I experienced normal daily activities as stressful. | | | | | | sometimes | |  |

All items had a time frame of the last 7 days

Example 11


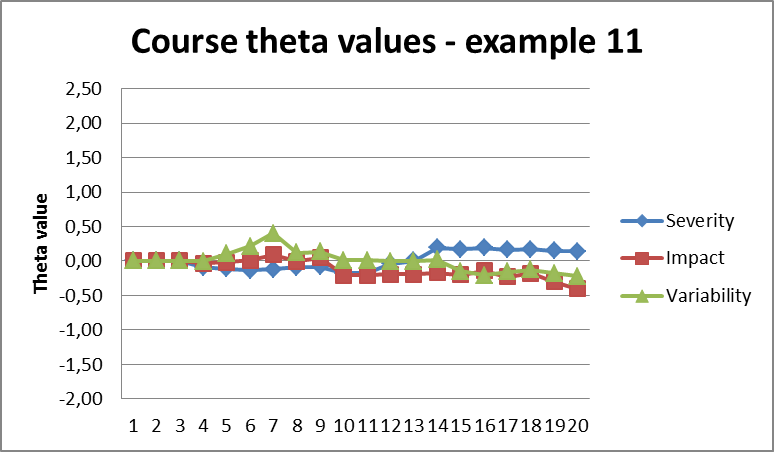


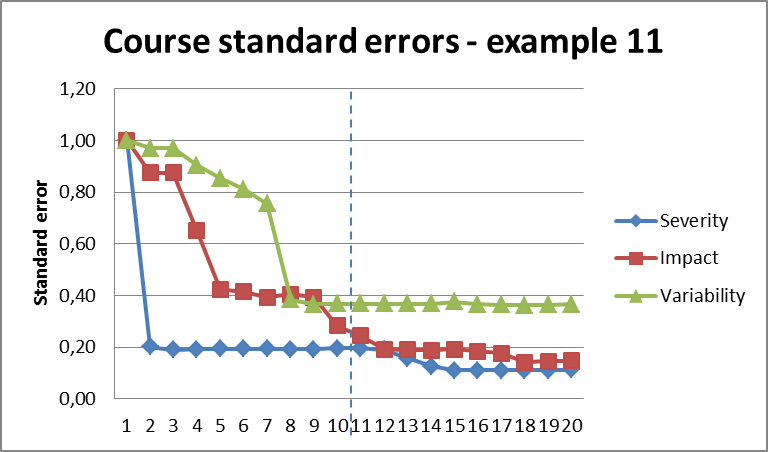


| item | dimension | | | theta d1 | theta d2 | theta d3 | SE d1 | SE d2 | SE d3 |
| --- | --- | --- | --- | --- | --- | --- | --- | --- | --- |
| 1 | 1 | | | 0,0000 | 0,0000 | 0,0000 | 1,0000 | 1,0000 | 1,0000 |
| 2 | 1 | | | 0,0000 | 0,0000 | 0,0000 | 0,2012 | 0,8746 | 0,9703 |
| 3 | 2 | | | 0,0000 | 0,0000 | 0,0000 | 0,1891 | 0,8739 | 0,9701 |
| 4 | 2 | | | -0,0914 | -0,0342 | -0,0076 | 0,1924 | 0,6508 | 0,9037 |
| 5 | 3 | | | -0,1131 | -0,0154 | 0,1055 | 0,1930 | 0,4233 | 0,8527 |
| 6 | 3 | | | -0,1312 | 0,0121 | 0,2144 | 0,1937 | 0,4144 | 0,8107 |
| 7 | 3 | | | -0,1207 | 0,0909 | 0,4003 | 0,1933 | 0,3925 | 0,7556 |
| 8 | 3 | | | -0,0887 | -0,0005 | 0,1200 | 0,1919 | 0,4043 | 0,3828 |
| 9 | 2 | | | -0,0847 | 0,0438 | 0,1304 | 0,1918 | 0,3935 | 0,3650 |
| 10 | 2 | | | -0,1764 | -0,2100 | 0,0095 | 0,1955 | 0,2831 | 0,3685 |
| 11 | 2 | | | -0,1776 | -0,2100 | 0,0089 | 0,1955 | 0,2442 | 0,3681 |
| 12 | 1 | | | -0,0390 | -0,1954 | -0,0044 | 0,1897 | 0,1911 | 0,3682 |
| 13 | 1 | | | 0,0064 | -0,1921 | -0,0069 | 0,1573 | 0,1906 | 0,3683 |
| 14 | 1 | | | 0,1885 | -0,1692 | 0,0178 | 0,1254 | 0,1875 | 0,3672 |
| 15 | 3 | | | 0,1674 | -0,1977 | -0,1507 | 0,1108 | 0,1914 | 0,3747 |
| 16 | 2 | | | 0,1881 | -0,1448 | -0,2078 | 0,1101 | 0,1843 | 0,3658 |
| 17 | 2 | | | 0,1642 | -0,2287 | -0,1583 | 0,1109 | 0,1761 | 0,3634 |
| 18 | 2 | | | 0,1708 | -0,1754 | -0,1295 | 0,1107 | 0,1408 | 0,3618 |
| 19 | 2 | | | 0,1512 | -0,3043 | -0,1799 | 0,1114 | 0,1449 | 0,3642 |
| 20 | 2 | | | 0,1404 | -0,4044 | -0,2230 | 0,1117 | 0,1464 | 0,3663 |
| item | dim. | nr. | itemstam | | | | | | answer |
| 1 | 1 | 5 | Did you feel tired? | | | | | | sometimes |
| 2 | 1 | 12 | How long, on average, has each episode of fatigue lasted? | | | | | | several hours |
| 3 | 2 | 87 | Fatigue made me feel less confident. | | | | | | not at all |
| 4 | 2 | 130 | Have you avoided making plans because of fatigue? e.g. plans to go out, or do jobs around the home or garden. | | | | | | a little |
| 5 | 3 | 185 | Could it vary how often you were fatigued? | | | | | | yes |
| 6 | 3 | 184 | The duration of my fatigue could vary. | | | | | | yes |
| 7 | 3 | 191 | Rheumatoid arthritis is the cause of my fatigue. | | | | | | yes |
| 8 | 3 | 189 | Performance of routine daily activities increases my fatigue. | | | | | | no |
| 9 | 2 | 138 | I really didn’t look forward to doing something due to the fatigue. | | | | | | a little |
| 10 | 2 | 150 | Fatigue meant I was less able to perform well. | | | | | | not at all |
| 11 | 2 | 38 | The fatigue felt like a physical burden. | | | | | | a little |
| 12 | 1 | 10 | I was even tired if I hadn’t done anything special | | | | | | sometimes |
| 13 | 1 | 9 | How often have you been fatigued? | | | | | | most but not all days |
| 14 | 1 | 2 | I felt tired. | | | | | | rather |
| 15 | 3 | 196 | My medication for rheumatism is the cause of my fatigue. | | | | | | no |
| 16 | 2 | 134 | I had to make choices which things I would or would not do, because I was too tired to do everything. | | | | | | a little |
| 17 | 2 | 167 | Because of my fatigue, I was less able to complete tasks that require physical effort. | | | | | | rarely |
| 18 | 2 | 135 | Fatigue made it difficult to undertake anything new. | | | | | | to a great extent |
| 19 | 2 | 104 | Fatigue meant that everything I did was such an effort. | | | | | | not at all |
| 20 | 2 | 33 | Physically I felt I was in a bad condition. | | | | | | rarely |

All items had a time frame of the last 7 days

Example 13


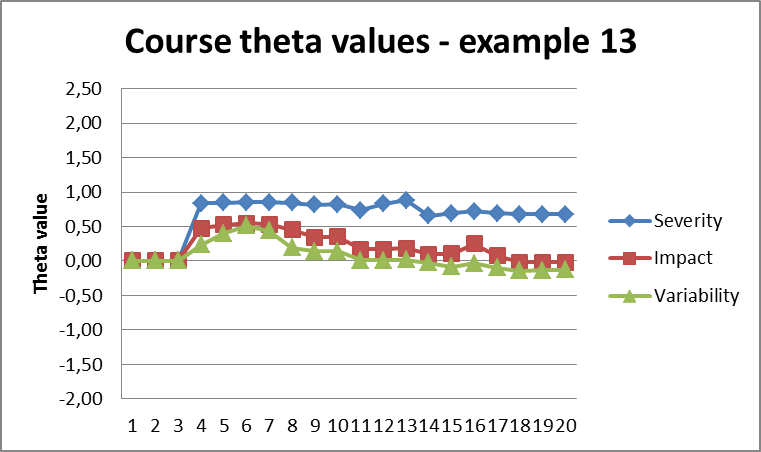


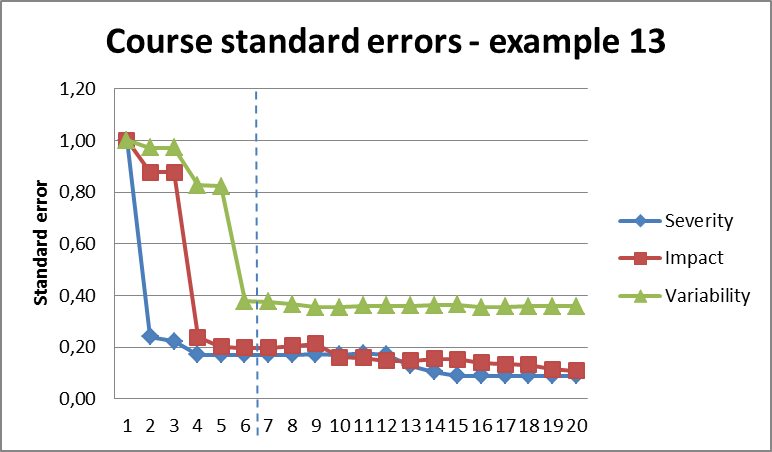


| item | dimension | | | theta d1 | theta d2 | theta d3 | SE d1 | SE d2 | | SE d3 |  |
| --- | --- | --- | --- | --- | --- | --- | --- | --- | --- | --- | --- |
| 1 | 1 | | | 0,0000 | 0,0000 | 0,0000 | 1,0000 | 1,0000 | | 1,0000 |  |
| 2 | 1 | | | 0,0000 | 0,0000 | 0,0000 | 0,2393 | 0,8769 | | 0,9708 |  |
| 3 | 2 | | | 0,0000 | 0,0000 | 0,0000 | 0,2221 | 0,8758 | | 0,9706 |  |
| 4 | 2 | | | 0,8382 | 0,4760 | 0,2332 | 0,1705 | 0,2384 | | 0,8261 |  |
| 5 | 3 | | | 0,8457 | 0,5245 | 0,4013 | 0,1702 | 0,2007 | | 0,8224 |  |
| 6 | 3 | | | 0,8490 | 0,5502 | 0,5145 | 0,1700 | 0,1970 | | 0,3770 |  |
| 7 | 3 | | | 0,8485 | 0,5359 | 0,4388 | 0,1700 | 0,1981 | | 0,3751 |  |
| 8 | 3 | | | 0,8440 | 0,4520 | 0,1904 | 0,1702 | 0,2047 | | 0,3653 |  |
| 9 | 2 | | | 0,8180 | 0,3436 | 0,1422 | 0,1714 | 0,2138 | | 0,3549 |  |
| 10 | 2 | | | 0,8203 | 0,3527 | 0,1428 | 0,1713 | 0,1615 | | 0,3546 |  |
| 11 | 2 | | | 0,7338 | 0,1695 | 0,0092 | 0,1754 | 0,1580 | | 0,3601 |  |
| 12 | 1 | | | 0,8269 | 0,1734 | 0,0113 | 0,1710 | 0,1473 | | 0,3599 |  |
| 13 | 1 | | | 0,8824 | 0,1812 | 0,0140 | 0,1279 | 0,1466 | | 0,3598 |  |
| 14 | 1 | | | 0,6574 | 0,0991 | -0,0257 | 0,1045 | 0,1541 | | 0,3616 |  |
| 15 | 3 | | | 0,6915 | 0,1029 | -0,0865 | 0,0890 | 0,1538 | | 0,3643 |  |
| 16 | 2 | | | 0,7196 | 0,2532 | -0,0305 | 0,0883 | 0,1405 | | 0,3537 |  |
| 17 | 2 | | | 0,6910 | 0,0741 | -0,1009 | 0,0890 | 0,1346 | | 0,3569 |  |
| 18 | 2 | | | 0,6793 | -0,0213 | -0,1404 | 0,0893 | 0,1321 | | 0,3588 |  |
| 19 | 2 | | | 0,6780 | -0,0166 | -0,1311 | 0,0893 | 0,1148 | | 0,3582 |  |
| 20 | 2 | | | 0,6762 | -0,0173 | -0,1264 | 0,0894 | 0,1089 | | 0,3580 |  |
| item | dim. | nr. | itemstam | | | | | | answer | | |
| 1 | 1 | 2 | I felt tired. | | | | | | rather | | |
| 2 | 1 | 4 | Fatigue was the worst consequence of my rheumatism. | | | | | | 5 strongly agree | | |
| 3 | 2 | 36 | My fatigue gets me down. | | | | | | sometimes | | |
| 4 | 2 | 59 | It took a lot of effort to keep focused when I was tired. | | | | | | a little | | |
| 5 | 3 | 191 | Rheumatoid arthritis is the cause of my fatigue. | | | | | | yes | | |
| 6 | 3 | 185 | Could it vary how often you were fatigued? | | | | | | yes | | |
| 7 | 3 | 189 | Performance of routine daily activities increases my fatigue. | | | | | | I don’t know | | |
| 8 | 3 | 196 | My medication for rheumatism is the cause of my fatigue. | | | | | | no | | |
| 9 | 2 | 138 | I really didn’t look forward to doing something due to the fatigue. | | | | | | a little | | |
| 10 | 2 | 150 | Fatigue meant I was less able to perform well. | | | | | | a little | | |
| 11 | 2 | 114 | I had to limit my social activities because I was tired. | | | | | | not at all | | |
| 12 | 1 | 3 | I felt fatigued. | | | | | | rather | | |
| 13 | 1 | 8 | Did you feel fatigued? | | | | | | usually | | |
| 14 | 1 | 5 | Did you feel tired? | | | | | | sometimes | | |
| 15 | 3 | 186 | The severity of my fatigue could vary. | | | | | | no | | |
| 16 | 2 | 38 | The fatigue felt like a physical burden. | | | | | | rather | | |
| 17 | 2 | 134 | I had to make choices which things I would or would not do, because I was too tired to do everything. | | | | | | not at all | | |
| 18 | 2 | 167 | Because of fatigue, I was less able to complete tasks that require physical effort. | | | | | | rarely | | |
| 19 | 2 | 104 | Fatigue meant that everything I did was such an effort. | | | | | | a little | | |
| 20 | 2 | 35 | I felt weak due to my fatigue. | | | | | | a little | | |

All items had a time frame of the last 7 days

Example 14


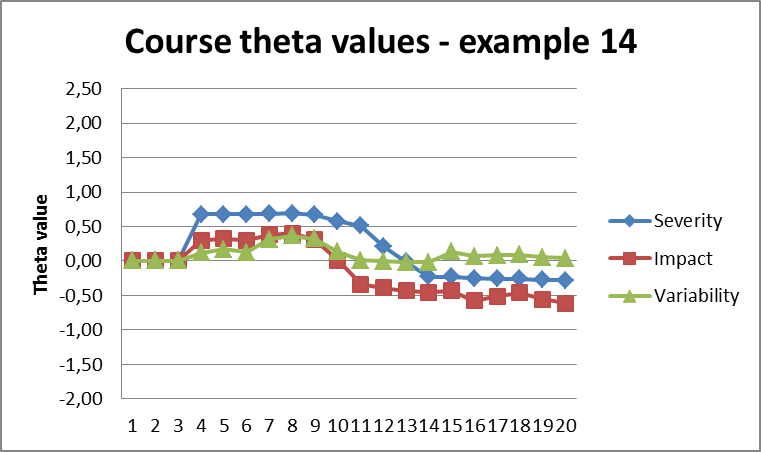


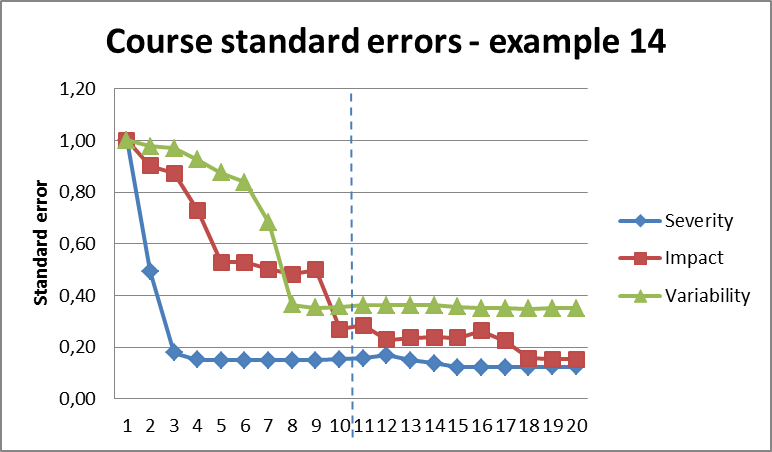


| item | | dimension | | | theta d1 | theta d2 | theta d3 | SE d1 | SE d2 | | SE d3 |
| --- | --- | --- | --- | --- | --- | --- | --- | --- | --- | --- | --- |
| 1 | | 1 | | | 0,0000 | 0,0000 | 0,0000 | 1,0000 | 1,0000 | | 1,0000 |
| 2 | | 1 | | | 0,0000 | 0,0000 | 0,0000 | 0,4939 | 0,9026 | | 0,9767 |
| 3 | | 2 | | | 0,0000 | 0,0000 | 0,0000 | 0,1782 | 0,8734 | | 0,9700 |
| 4 | | 2 | | | 0,6751 | 0,3006 | 0,1211 | 0,1502 | 0,7269 | | 0,9248 |
| 5 | | 3 | | | 0,6778 | 0,3187 | 0,1673 | 0,1499 | 0,5279 | | 0,8739 |
| 6 | | 3 | | | 0,6755 | 0,3012 | 0,1241 | 0,1500 | 0,5286 | | 0,8383 |
| 7 | | 3 | | | 0,6875 | 0,3812 | 0,3168 | 0,1496 | 0,5020 | | 0,6807 |
| 8 | | 3 | | | 0,6905 | 0,4048 | 0,3666 | 0,1494 | 0,4829 | | 0,3632 |
| 9 | | 2 | | | 0,6719 | 0,3108 | 0,3255 | 0,1501 | 0,4988 | | 0,3521 |
| 10 | | 2 | | | 0,5777 | 0,0129 | 0,1306 | 0,1536 | 0,2684 | | 0,3564 |
| 11 | | 2 | | | 0,5077 | -0,3409 | 0,0137 | 0,1563 | 0,2854 | | 0,3615 |
| 12 | | 1 | | | 0,2148 | -0,3913 | -0,0019 | 0,1682 | 0,2284 | | 0,3616 |
| 13 | | 1 | | | -0,0119 | -0,4318 | -0,0169 | 0,1492 | 0,2354 | | 0,3624 |
| 14 | | 1 | | | -0,2310 | -0,4515 | -0,0160 | 0,1367 | 0,2389 | | 0,3623 |
| 15 | | 3 | | | -0,2308 | -0,4324 | 0,1304 | 0,1218 | 0,2355 | | 0,3561 |
| 16 | | 2 | | | -0,2538 | -0,5803 | 0,0692 | 0,1228 | 0,2637 | | 0,3508 |
| 17 | | 2 | | | -0,2589 | -0,5136 | 0,0832 | 0,1229 | 0,2249 | | 0,3498 |
| 18 | | 2 | | | -0,2591 | -0,4604 | 0,0914 | 0,1229 | 0,1574 | | 0,3490 |
| 19 | | 2 | | | -0,2730 | -0,5584 | 0,0553 | 0,1235 | 0,1528 | | 0,3505 |
| 20 | | 2 | | | -0,2789 | -0,6101 | 0,0417 | 0,1237 | 0,1521 | | 0,3511 |
| item | dim. | | nr. | itemstam | | | | | | answer | |
| 1 | 1 | | 7 | I was particularly tired in the evening. | | | | | | 5-6 days | |
| 2 | 1 | | 8 | Did you feel fatigued? | | | | | | usually | |
| 3 | 2 | | 67 | Have you made mistakes because of fatigue? | | | | | | not at all | |
| 4 | 2 | | 74 | My fatigue soon made me irritated. | | | | | | a little | |
| 5 | 3 | | 187 | Exercise brings on my fatigue. | | | | | | I don’t know | |
| 6 | 3 | | 189 | Performance of routine daily activities increases my fatigue. | | | | | | I don’t know | |
| 7 | 3 | | 191 | Rheumatoid arthritis is the cause of my fatigue. | | | | | | yes | |
| 8 | 3 | | 196 | My medication for rheumatism is the cause of my fatigue. | | | | | | I don’t know | |
| 9 | 2 | | 138 | I really didn’t look forward to doing something due to the fatigue. | | | | | | a little | |
| 10 | 2 | | 150 | Fatigue meant I was less able to perform well. | | | | | | not at all | |
| 11 | 2 | | 38 | The fatigue felt like a physical burden. | | | | | | not at all | |
| 12 | 1 | | 3 | I felt fatigued. | | | | | | a little | |
| 13 | 1 | | 2 | I felt tired. | | | | | | a little | |
| 14 | 1 | | 9 | How often have you been fatigued? | | | | | | occasionally but not most days | |
| 15 | 3 | | 186 | The severity of my fatigue could vary. | | | | | | yes | |
| 16 | 2 | | 134 | I had to make choices which things I would or would not do, because I was too tired to do everything. | | | | | | not at all | |
| 17 | 2 | | 33 | Physically I felt I was in a bad condition. | | | | | | sometimes | |
| 18 | 2 | | 145 | Fatigue meant it took longer to finish things. | | | | | | a little | |
| 19 | 2 | | 135 | Fatigue made it difficult to undertake anything new. | | | | | | not at all | |
| 20 | 2 | | 164 | Fatigue prevented me from physically exerting myself for a longer period of time. | | | | | | rarely | |

All items had a time frame of the last 7 days

Example 15


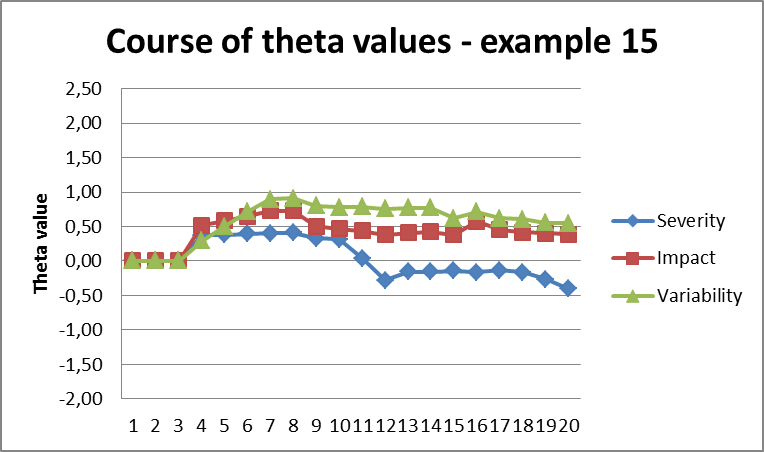


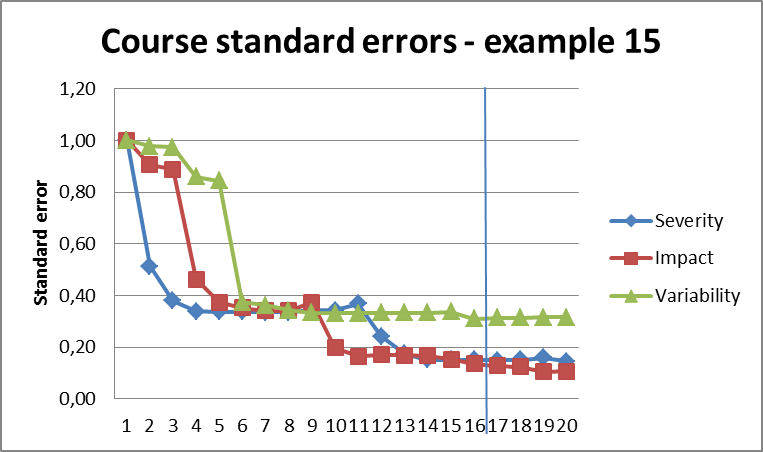


| item | | dimension | | | theta d1 | theta d2 | theta d3 | SE d1 | SE d2 | | SE d3 |
| --- | --- | --- | --- | --- | --- | --- | --- | --- | --- | --- | --- |
| 1 | | 1 | | | 0,0000 | 0,0000 | 0,0000 | 1,0000 | 1,0000 | | 1,0000 |
| 2 | | 1 | | | 0,0000 | 0,0000 | 0,0000 | 0,5121 | 0,9051 | | 0,9772 |
| 3 | | 2 | | | 0,0000 | 0,0000 | 0,0000 | 0,3803 | 0,8891 | | 0,9736 |
| 4 | | 2 | | | 0,3701 | 0,5207 | 0,2955 | 0,3390 | 0,4625 | | 0,8599 |
| 5 | | 3 | | | 0,3833 | 0,5847 | 0,5026 | 0,3372 | 0,3728 | | 0,8439 |
| 6 | | 3 | | | 0,3965 | 0,6521 | 0,7168 | 0,3361 | 0,3520 | | 0,3737 |
| 7 | | 3 | | | 0,4049 | 0,7286 | 0,8987 | 0,3353 | 0,3408 | | 0,3628 |
| 8 | | 3 | | | 0,4058 | 0,7321 | 0,9122 | 0,3352 | 0,3400 | | 0,3429 |
| 9 | | 2 | | | 0,3291 | 0,5062 | 0,7987 | 0,3420 | 0,3731 | | 0,3330 |
| 10 | | 2 | | | 0,3157 | 0,4655 | 0,7821 | 0,3422 | 0,1978 | | 0,3320 |
| 11 | | 1 | | | 0,0374 | 0,4378 | 0,7870 | 0,3682 | 0,1648 | | 0,3316 |
| 12 | | 1 | | | -0,2770 | 0,3803 | 0,7543 | 0,2426 | 0,1703 | | 0,3326 |
| 13 | | 1 | | | -0,1586 | 0,4079 | 0,7721 | 0,1737 | 0,1676 | | 0,3321 |
| 14 | | 2 | | | -0,1537 | 0,4203 | 0,7719 | 0,1512 | 0,1664 | | 0,3321 |
| 15 | | 3 | | | -0,1405 | 0,3816 | 0,6210 | 0,1504 | 0,1530 | | 0,3363 |
| 16 | | 2 | | | -0,1636 | 0,5782 | 0,7120 | 0,1518 | 0,1359 | | 0,3104 |
| 17 | | 2 | | | -0,1341 | 0,4535 | 0,6169 | 0,1501 | 0,1272 | | 0,3130 |
| 18 | | 2 | | | -0,1625 | 0,4195 | 0,6086 | 0,1517 | 0,1237 | | 0,3133 |
| 19 | | 1 | | | -0,2649 | 0,4028 | 0,5570 | 0,1580 | 0,1050 | | 0,3148 |
| 20 | | 1 | | | -0,4065 | 0,3906 | 0,5490 | 0,1460 | 0,1057 | | 0,3150 |
| item | dim. | | nr. | itemstam | | | | | | answer | |
| 1 | 1 | | 4 | Fatigue was the worst consequence of my rheumatism. | | | | | | 2 | |
| 2 | 1 | | 7 | I was particularly tired in the evening. | | | | | | every day | |
| 3 | 2 | | 25 | I needed to go to bed early in order to be able to function. | | | | | | always | |
| 4 | 2 | | 159 | I was too tired to talk. | | | | | | never | |
| 5 | 3 | | 191 | Rheumatoid arthritis is the cause of my fatigue. | | | | | | yes | |
| 6 | 3 | | 190 | Stress brings on my fatigue. | | | | | | yes | |
| 7 | 3 | | 189 | Performance of routine daily activities increases my fatigue. | | | | | | yes | |
| 8 | 3 | | 196 | My medication for rheumatism is the cause of my fatigue. | | | | | | I don’t know | |
| 9 | 2 | | 138 | I really didn’t look forward to doing something due to the fatigue. | | | | | | a little | |
| 10 | 2 | | 150 | Fatigue meant I was less able to perform well. | | | | | | a little | |
| 11 | 1 | | 3 | I felt fatigued. | | | | | | a little | |
| 12 | 1 | | 9 | How often have you been fatigued? | | | | | | occasionally but not most days | |
| 13 | 1 | | 10 | I was even tired if I hadn’t done anything special. | | | | | | sometimes | |
| 14 | 2 | | 114 | I had to limit my social activities because I was tired. | | | | | | a little | |
| 15 | 3 | | 183 | To what degree has your fatigue changed? | | | | | | stayed the same | |
| 16 | 2 | | 134 | I had to make choices which things I would or would not do, because I was too tired to do everything. | | | | | | rather | |
| 17 | 2 | | 149 | I was too tired to do my most important tasks. | | | | | | not at all | |
| 18 | 2 | | 167 | Because of my fatigue, I was less able to complete tasks that require physical effort. | | | | | | sometimes | |
| 19 | 1 | | 1 | Please circle the number that shows your average level of fatigue. | | | | | | 1 | |
| 20 | 1 | | 6 | How many days did you experience fatigue? | | | | | | 2 | |

All items had a time frame of the last 7 days
